# Correction: Introgression of the SbASR-1 Gene Cloned from a Halophyte *Salicornia brachiata* Enhances Salinity and Drought Endurance in Transgenic Groundnut (*Arachis hypogaea*) and Acts as a Transcription Factor

**DOI:** 10.1371/journal.pone.0135541

**Published:** 2015-08-07

**Authors:** The PLOS ONE Staff

## Notice of Republication

This article was republished on July 22, 2015, to correct an error in the title introduced during the typesetting process. The publisher apologizes for the error. Please download this article again to view the correct version. The originally published, uncorrected article and the republished, corrected article are provided here for reference.

## Supporting Information

S1 File Originally published, uncorrected article. (PDF)

S2 File Republished, corrected article. (PDF)
