# Supplementary material for: Introgression of the SbASR-1 Gene Cloned from a Halophyte Salicornia brachiata Enhances Salinity and Drought Endurance in Transgenic Groundnut (Arachis hypogaea) and Acts as a Transcription Factor
Source: PLoS One. 2015 Jul 9;10(7):e0131567. doi: 10.1371/journal.pone.0131567 (PMC4497679; doi:10.1371/journal.pone.0131567)
Supplement: S1 Table — (DOC) [file pone.0131567.s001.doc]

**Supplementary Table S1: Primers used in the study and PCR conditions**

| **Purpose** | **Primer** | **Orientation** | **Sequence (5’-3’)** | **PCR conditions** |
| --- | --- | --- | --- | --- |
| Isolation of  promoter | AR-f | Reverse | GCACAGGGCCCTGGTCTTCA | As suggested in Universal Genome Walker Kit manual |
|  | AR-e | Reverse | CATGCCGGGTAGGTGTGCAAG |  |
|  | AR-d | Reverse | TGCATACATACCACGTAACTACG |  |
|  | AR-c | Reverse | GTTGAACCATAGCCTCCTCTTTC |  |
|  | AR-b | Reverse | CACCGGTGTCGGAGCCATAGC |  |
|  | AR-a | Reverse | ACCAGTCTCGGTGGAGCCATAG |  |
| Full length promoter | ARPF | Forward | GGGATTAGTAGCGGAAGATGG | [95°C-5min] x1; [95°C-15sec, 60°C-30sec, 68°C-2min] x35; [68°C-7min] x1 |
|  | ARPR | Reverse | GAATAATTAAAGTGATTTGAATGATG |  |
| Confirmation of transgenic lines and probe for Southern hybridization | ASRF | Forward | CCGAGACCAAGAGGTATGG | [95°C-3min] x1; [95°C-30sec, 60°C-30sec, 72°C-40sec] x30; [72°C-7min] x1 |
|  | ASRR | Reverse | GGGAATTCTTAGAAGAAATGGTGCTTCTTTTCC |  |
| Cloning of *Sb*ASR-1 into pET28a expression vector | PARF | Forward | GAGCTCATGGAAAGAGGAGGCTATGGTTCAACCG | [95°C-3min] x1; [95°C-30sec, 60°C-30sec, 72°C-40sec] x30; [72°C-7min] x1 |
|  | PARR | Reverse | AAGCTTTTAGAAGAAATGGTGCTTCTTTTCC |  |
| Genomic organization and copy number | ASRP | Forward | CGTAGTTACGTGGTATGTATGC | [95°C-5min] x1; [95°C-15sec, 60°C-30sec, 68°C-3min] x30; [68°C-7min] x1 |
|  | ASRR | Reverse | AAGCTTTTAGAAGAAATGGTGCTTCTTTTCC |  |
| Transcript expression of antioxidative enzyme genes | AhAPXF | Forward | TGCTGGAACTTTTGATGTGG | [95°C-5min] x1; [95°C-10sec,  60°C-30sec] x40 followed by melt curve analysis |
|  | AhAPXR | Forward | AACTACACCGGCCAACTG |  |
|  | AhCATF | Forward | TTTTACACCAGAGAGGGTAACT |  |
|  | AhCATR | Forward | AGGATCCTCCAATTCTCCTGG |  |
|  | AhSODF | Forward | CAGTTCTTAGCAGCAGTGAG |  |
|  | AhSODR | Reverse | GGAACCCATGAAGACCAG |  |
|  | AhACTF | Forward | CGGGATGGAATCTCCTGGA |  |
|  | AhACTR | Reverse | CATGCTACTCGGTGCCAATG |  |
| Subcellular localization | ASRLoF | Forward | CACCATGGAAAGAGGAGGCTATGG | [95°C-5min] x1; [95°C-30sec, 60°C-30sec, 72°C-60sec] x35; [72°C-7min] x1 |
|  | ASRLoR | Reverse | TTAGAAGAAATGGTGCTTCTTTTC |  |
