# Supplementary material for: Introgression of the SbASR-1 Gene Cloned from a Halophyte Salicornia brachiata Enhances Salinity and Drought Endurance in Transgenic Groundnut (Arachis hypogaea) and Acts as a Transcription Factor
Source: PLoS One. 2015 Jul 9;10(7):e0131567. doi: 10.1371/journal.pone.0131567 (PMC4497679; doi:10.1371/journal.pone.0131567)
Supplement: S1 Fig — The probe sequences were adopted from the Rom et al. (2006) and their consensus sequence were modified. For probe ARBS-1 to ARBS-4, consensus sequence were adopted from Rom et al. (2006), while for ARBS-5 to ARBS-8 adopted from Ricardi et al. (2014). (PPTX) [file pone.0131567.s003.pptx]

## Slide 1
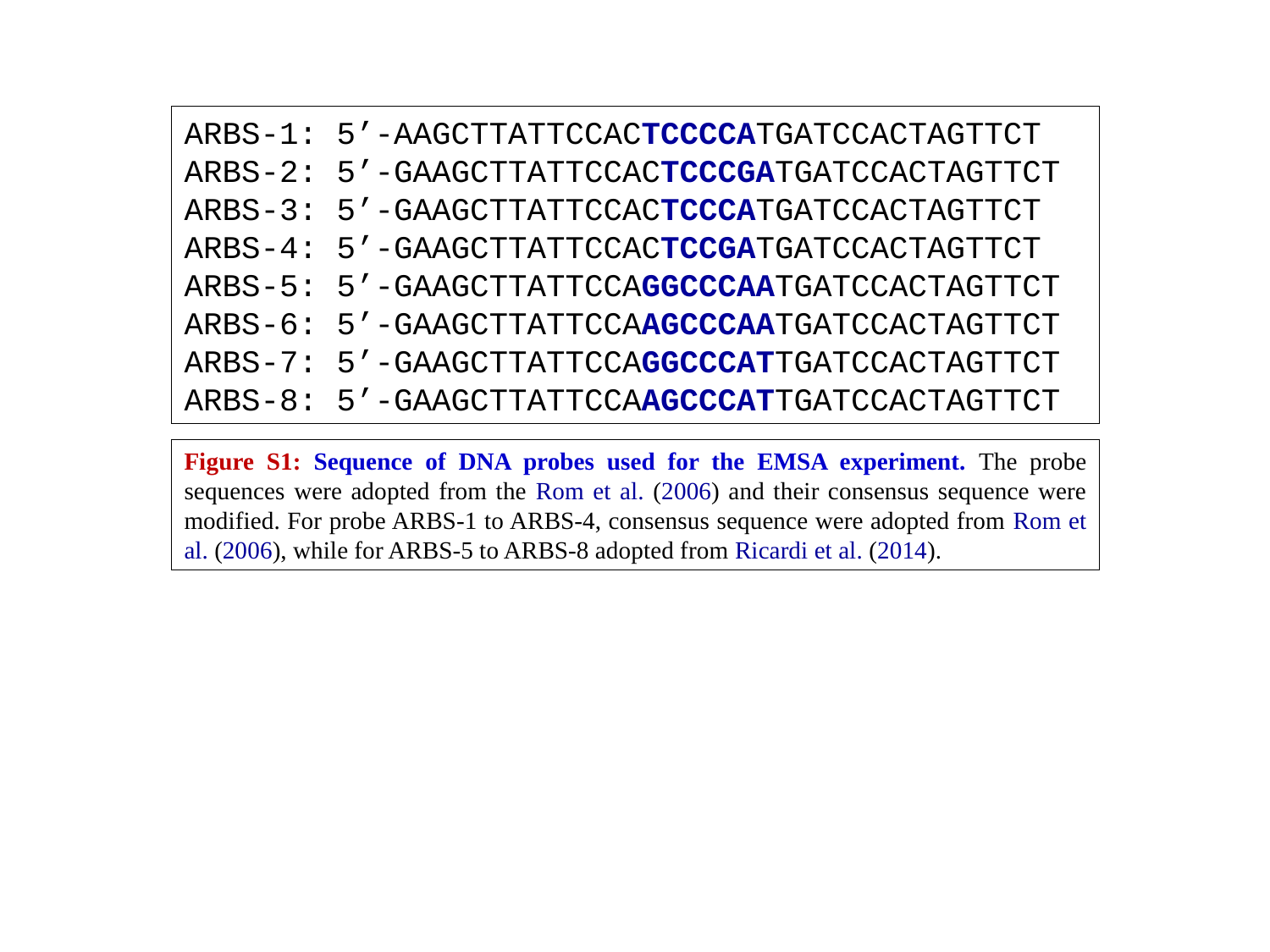

ARBS-1: 5’-AAGCTTATTCCACTCCCCATGATCCACTAGTTCT
ARBS-2: 5’-GAAGCTTATTCCACTCCCGATGATCCACTAGTTCT
ARBS-3: 5’-GAAGCTTATTCCACTCCCATGATCCACTAGTTCT
ARBS-4: 5’-GAAGCTTATTCCACTCCGATGATCCACTAGTTCT
ARBS-5: 5’-GAAGCTTATTCCAGGCCCAATGATCCACTAGTTCT
ARBS-6: 5’-GAAGCTTATTCCAAGCCCAATGATCCACTAGTTCT
ARBS-7: 5’-GAAGCTTATTCCAGGCCCATTGATCCACTAGTTCT
ARBS-8: 5’-GAAGCTTATTCCAAGCCCATTGATCCACTAGTTCT
Figure S1: Sequence of DNA probes used for the EMSA experiment. The probe sequences were adopted from the Rom et al. (2006) and their consensus sequence were modified. For probe ARBS-1 to ARBS-4, consensus sequence were adopted from Rom et al. (2006), while for ARBS-5 to ARBS-8 adopted from Ricardi et al. (2014).
