# Supplementary material for: Introgression of the SbASR-1 Gene Cloned from a Halophyte Salicornia brachiata Enhances Salinity and Drought Endurance in Transgenic Groundnut (Arachis hypogaea) and Acts as a Transcription Factor
Source: PLoS One. 2015 Jul 9;10(7):e0131567. doi: 10.1371/journal.pone.0131567 (PMC4497679; doi:10.1371/journal.pone.0131567)
Supplement: S2 Fig — Lane PC: Positive control SbASR-1 cloned vector; Lane E, H and X: Genomic DNA o digested with EcoRI, HindIII and XbaI enzymes, respectively. (PPTX) [file pone.0131567.s004.pptx]

## Slide 1
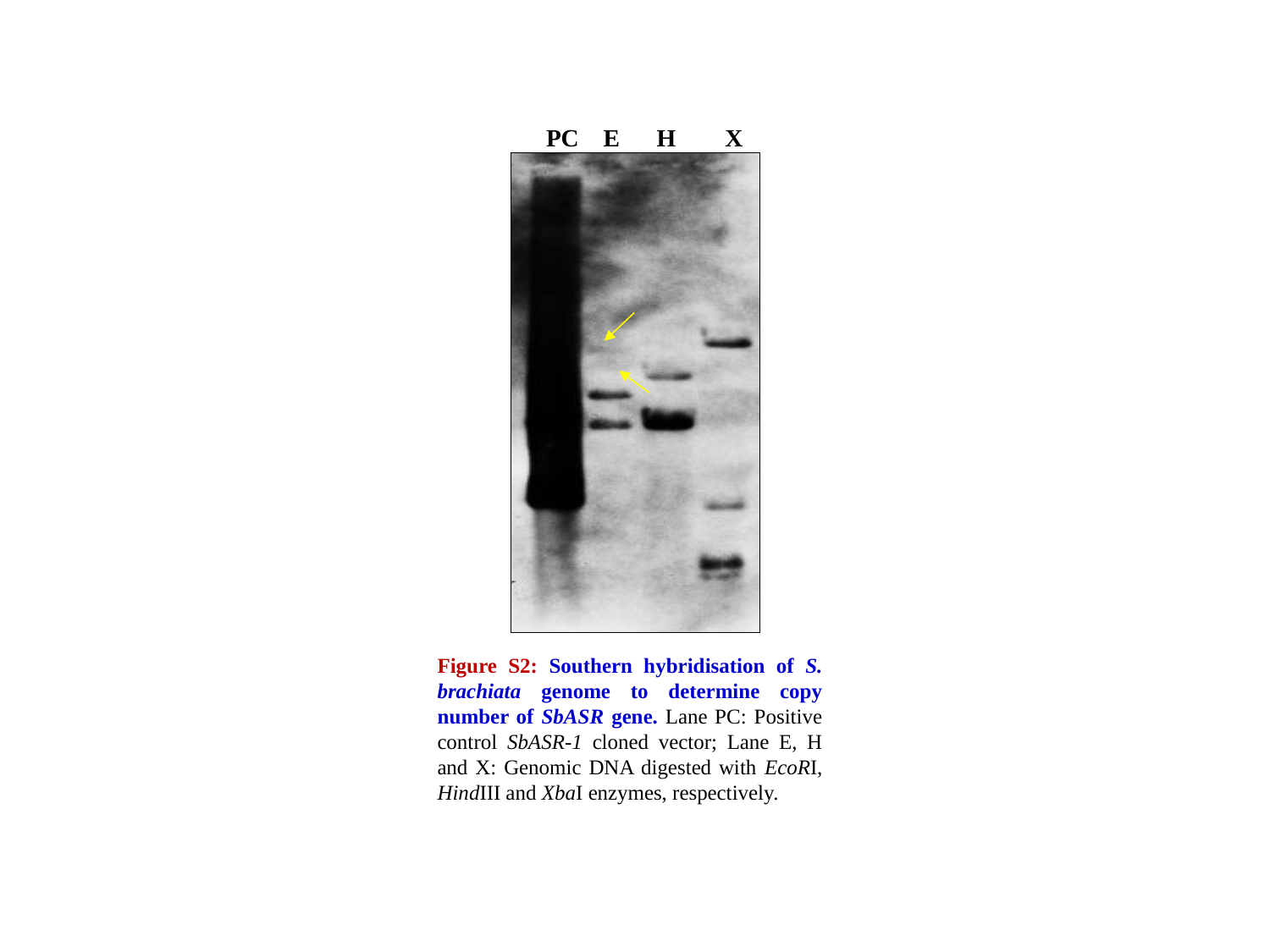

PC E H X
Figure S2: Southern hybridisation of S. brachiata genome to determine copy number of SbASR gene. Lane PC: Positive control SbASR-1 cloned vector; Lane E, H and X: Genomic DNA digested with EcoRI, HindIII and XbaI enzymes, respectively.
