# Supplementary material for: Introgression of the SbASR-1 Gene Cloned from a Halophyte Salicornia brachiata Enhances Salinity and Drought Endurance in Transgenic Groundnut (Arachis hypogaea) and Acts as a Transcription Factor
Source: PLoS One. 2015 Jul 9;10(7):e0131567. doi: 10.1371/journal.pone.0131567 (PMC4497679; doi:10.1371/journal.pone.0131567)
Supplement: S3 Fig — The underlined amino acid sequence shows the conserved domain of ABA/WDS protein super family. Four conserved domains 3, 1, 2 and 5 of LEA Group 7 proteins are displayed in green, red, blue and purple coloured fonts, respectively. The black coloured box in domain 1 represents the DNA binding domain. (PPTX) [file pone.0131567.s005.pptx]

## Slide 1
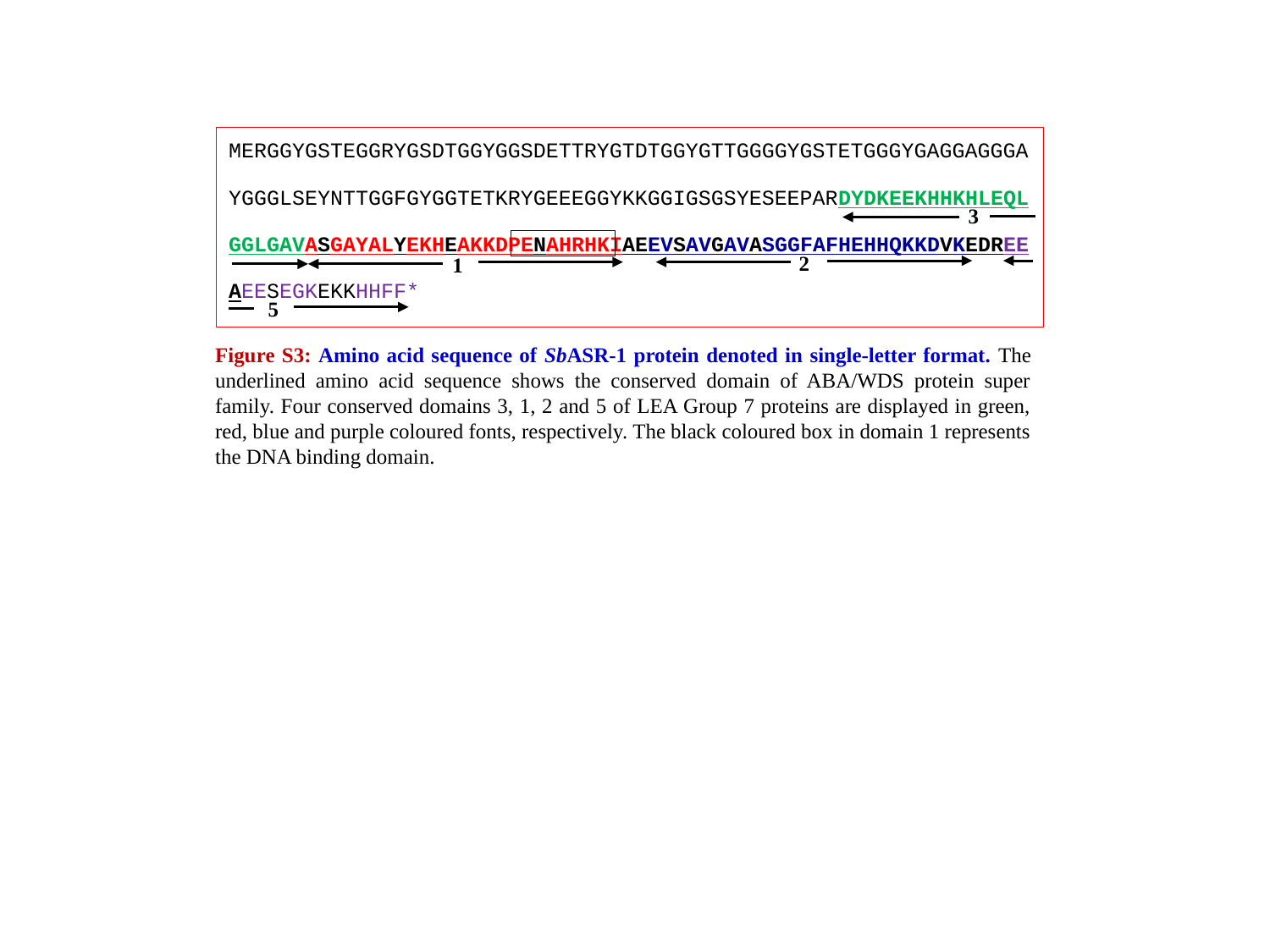

MERGGYGSTEGGRYGSDTGGYGGSDETTRYGTDTGGYGTTGGGGYGSTETGGGYGAGGAGGGA
YGGGLSEYNTTGGFGYGGTETKRYGEEEGGYKKGGIGSGSYESEEPARDYDKEEKHHKHLEQL
GGLGAVASGAYALYEKHEAKKDPENAHRHKIAEEVSAVGAVASGGFAFHEHHQKKDVKEDREE
AEESEGKEKKHHFF*
3
2
1
5
Figure S3: Amino acid sequence of SbASR-1 protein denoted in single-letter format. The underlined amino acid sequence shows the conserved domain of ABA/WDS protein super family. Four conserved domains 3, 1, 2 and 5 of LEA Group 7 proteins are displayed in green, red, blue and purple coloured fonts, respectively. The black coloured box in domain 1 represents the DNA binding domain.
