# Supplementary material for: Introgression of the SbASR-1 Gene Cloned from a Halophyte Salicornia brachiata Enhances Salinity and Drought Endurance in Transgenic Groundnut (Arachis hypogaea) and Acts as a Transcription Factor
Source: PLoS One. 2015 Jul 9;10(7):e0131567. doi: 10.1371/journal.pone.0131567 (PMC4497679; doi:10.1371/journal.pone.0131567)
Supplement: S4 Fig — Lane 1, protein standard marker; lane 2–4, BL21 (DE3); lane 5–7, BL21 containing pET28a; lane 8–10, BL21 harboring pET28a:SbASR-1 after induction with 1mM IPTG. (PPTX) [file pone.0131567.s006.pptx]

## Slide 1
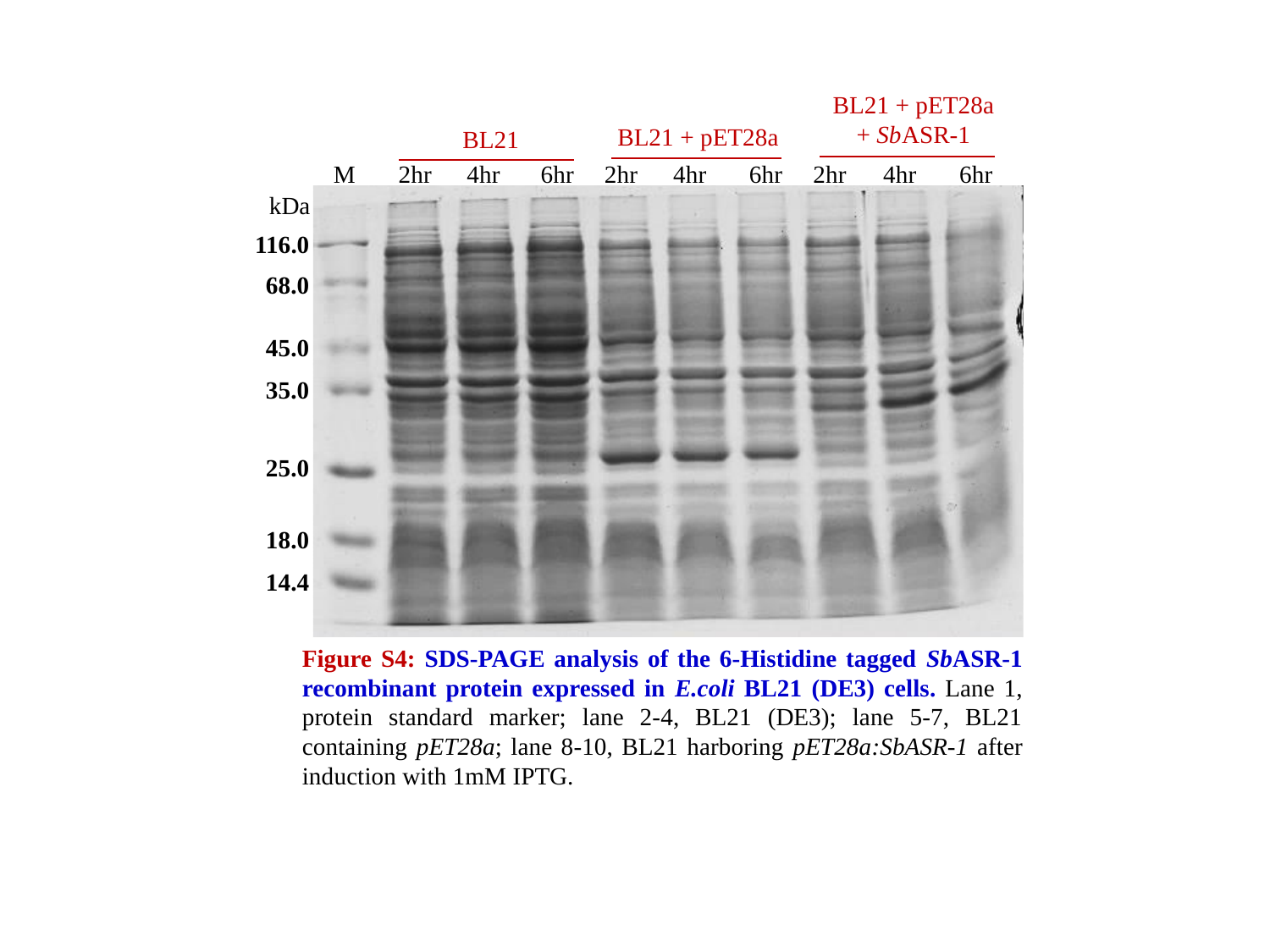

BL21 + pET28a
+ SbASR-1
BL21 + pET28a
BL21
M 2hr 4hr 6hr 2hr 4hr 6hr 2hr 4hr 6hr
kDa
116.0
68.0
45.0
35.0
25.0
18.0
14.4
Figure S4: SDS-PAGE analysis of the 6-Histidine tagged SbASR-1 recombinant protein expressed in E.coli BL21 (DE3) cells. Lane 1, protein standard marker; lane 2-4, BL21 (DE3); lane 5-7, BL21 containing pET28a; lane 8-10, BL21 harboring pET28a:SbASR-1 after induction with 1mM IPTG.
