# Supplementary material for: Introgression of the SbASR-1 Gene Cloned from a Halophyte Salicornia brachiata Enhances Salinity and Drought Endurance in Transgenic Groundnut (Arachis hypogaea) and Acts as a Transcription Factor
Source: PLoS One. 2015 Jul 9;10(7):e0131567. doi: 10.1371/journal.pone.0131567 (PMC4497679; doi:10.1371/journal.pone.0131567)
Supplement: S5 Fig — Lane 1 showed standard molecular marker of different size. Lane 2–6 have different fractions of elute (E1-E5). A light band of about 70 kDa appears due to dimerization of SbASR-1 protein. (PPTX) [file pone.0131567.s007.pptx]

## Slide 1
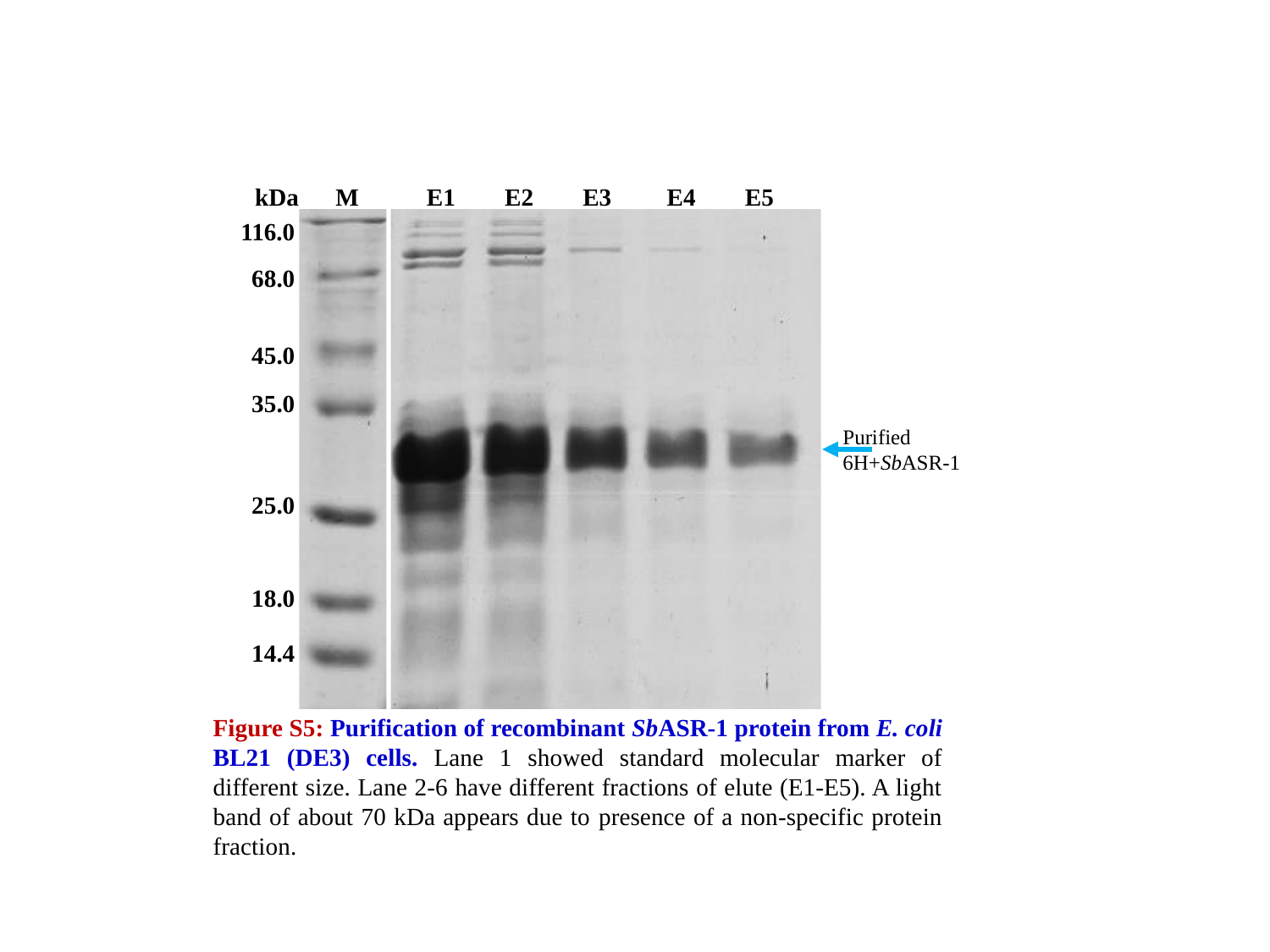

kDa M E1 E2 E3 E4 E5
116.0
68.0
45.0
35.0
25.0
18.0
14.4
Purified
6H+SbASR-1
Figure S5: Purification of recombinant SbASR-1 protein from E. coli BL21 (DE3) cells. Lane 1 showed standard molecular marker of different size. Lane 2-6 have different fractions of elute (E1-E5). A light band of about 70 kDa appears due to presence of a non-specific protein fraction.
