# Supplementary material for: Introgression of the SbASR-1 Gene Cloned from a Halophyte Salicornia brachiata Enhances Salinity and Drought Endurance in Transgenic Groundnut (Arachis hypogaea) and Acts as a Transcription Factor
Source: PLoS One. 2015 Jul 9;10(7):e0131567. doi: 10.1371/journal.pone.0131567 (PMC4497679; doi:10.1371/journal.pone.0131567)
Supplement: S6 Fig — All probes ARSB-1 to ARBS-8 showed clear shifting of band (denoted with blue arrow) which was absent in the lane containing only probe. (PPTX) [file pone.0131567.s008.pptx]

## Slide 1
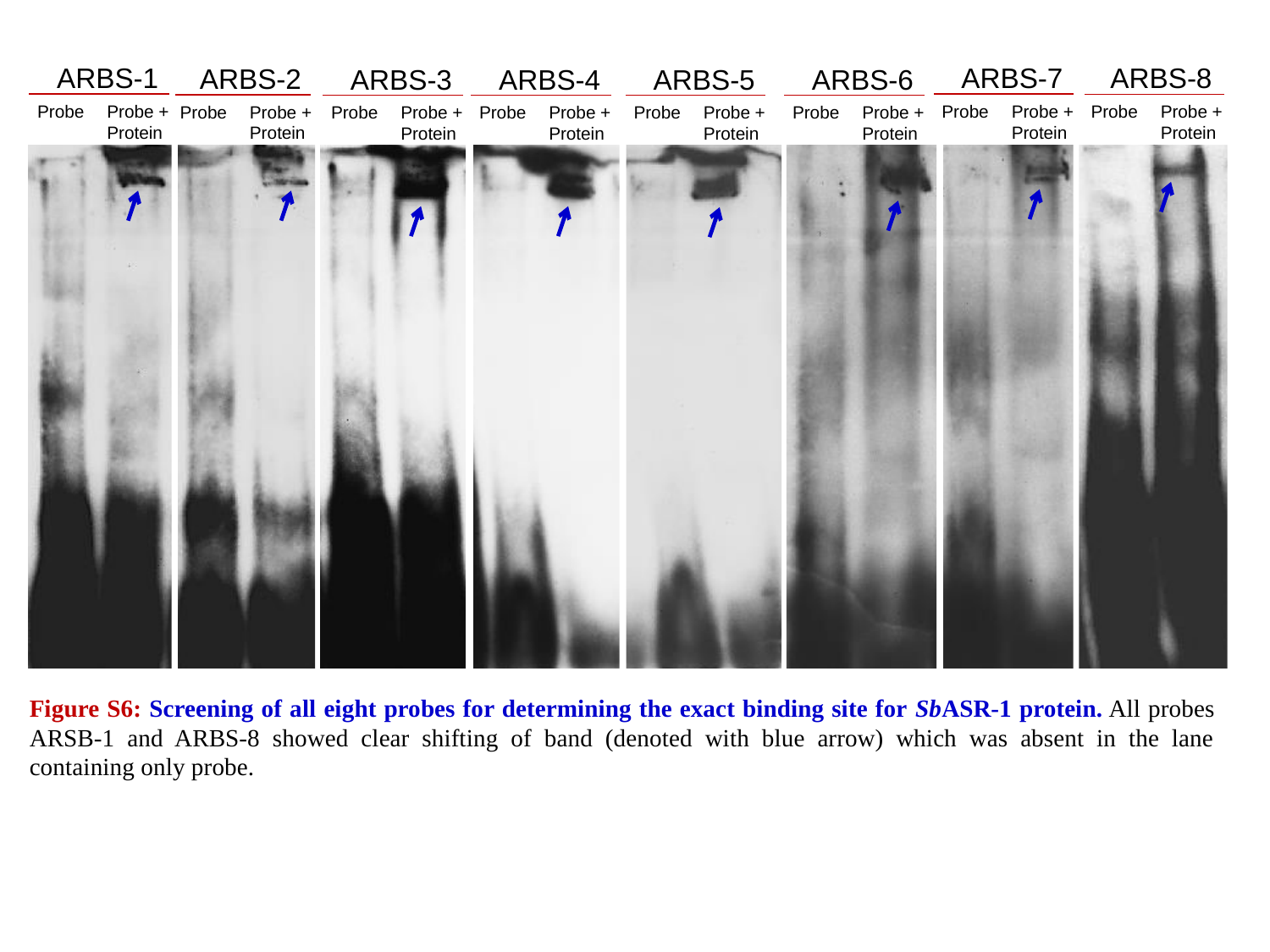

ARBS-1
Probe
Probe + Protein
ARBS-7
Probe
Probe + Protein
ARBS-8
Probe
Probe + Protein
ARBS-2
Probe
Probe + Protein
ARBS-3
Probe
Probe + Protein
ARBS-4
Probe
Probe + Protein
ARBS-5
Probe
Probe + Protein
ARBS-6
Probe
Probe + Protein
Figure S6: Screening of all eight probes for determining the exact binding site for SbASR-1 protein. All probes ARSB-1 and ARBS-8 showed clear shifting of band (denoted with blue arrow) which was absent in the lane containing only probe.
