# Supplementary material for: Introgression of the SbASR-1 Gene Cloned from a Halophyte Salicornia brachiata Enhances Salinity and Drought Endurance in Transgenic Groundnut (Arachis hypogaea) and Acts as a Transcription Factor
Source: PLoS One. 2015 Jul 9;10(7):e0131567. doi: 10.1371/journal.pone.0131567 (PMC4497679; doi:10.1371/journal.pone.0131567)
Supplement: S7 Fig — Amplification of SbASR-1 gene and Southern hybridization of Wt and different transgenic lines with SbASR-1 specific probe. Lane M: molecular marker, PC: positive control, Wt: wild type plant (non-transformed) and A1-A5: transgenic lines. (PPTX) [file pone.0131567.s009.pptx]

## Slide 1
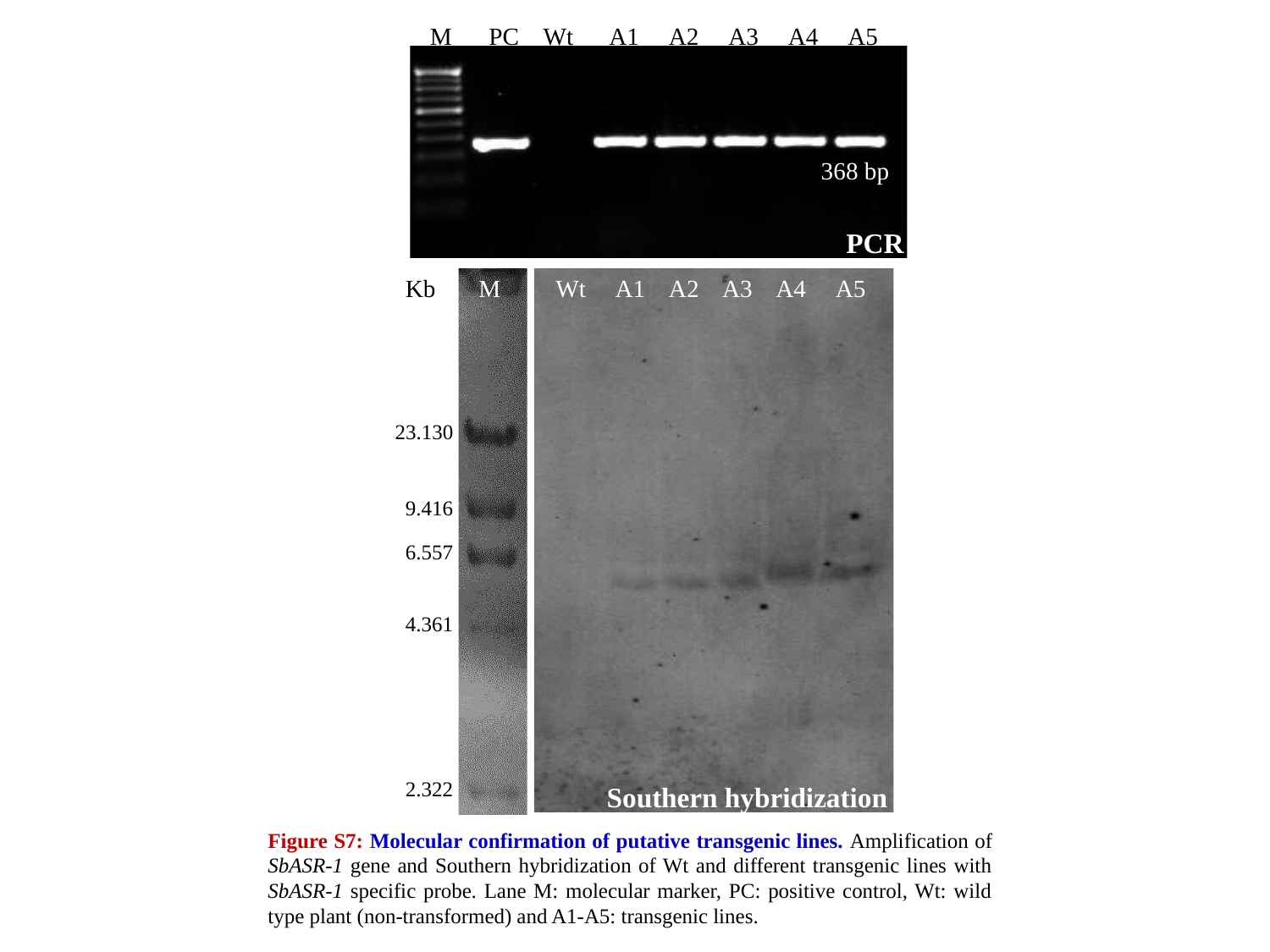

M PC Wt A1 A2 A3 A4 A5
368 bp
 Kb M Wt A1 A2 A3 A4 A5
23.130
9.416
6.557
4.361
2.322
PCR
Southern hybridization
Figure S7: Molecular confirmation of putative transgenic lines. Amplification of SbASR-1 gene and Southern hybridization of Wt and different transgenic lines with SbASR-1 specific probe. Lane M: molecular marker, PC: positive control, Wt: wild type plant (non-transformed) and A1-A5: transgenic lines.
