# Supplementary material for: Introgression of the SbASR-1 Gene Cloned from a Halophyte Salicornia brachiata Enhances Salinity and Drought Endurance in Transgenic Groundnut (Arachis hypogaea) and Acts as a Transcription Factor
Source: PLoS One. 2015 Jul 9;10(7):e0131567. doi: 10.1371/journal.pone.0131567 (PMC4497679; doi:10.1371/journal.pone.0131567)
Supplement: S8 Fig — (PPTX) [file pone.0131567.s010.pptx]

## Slide 1
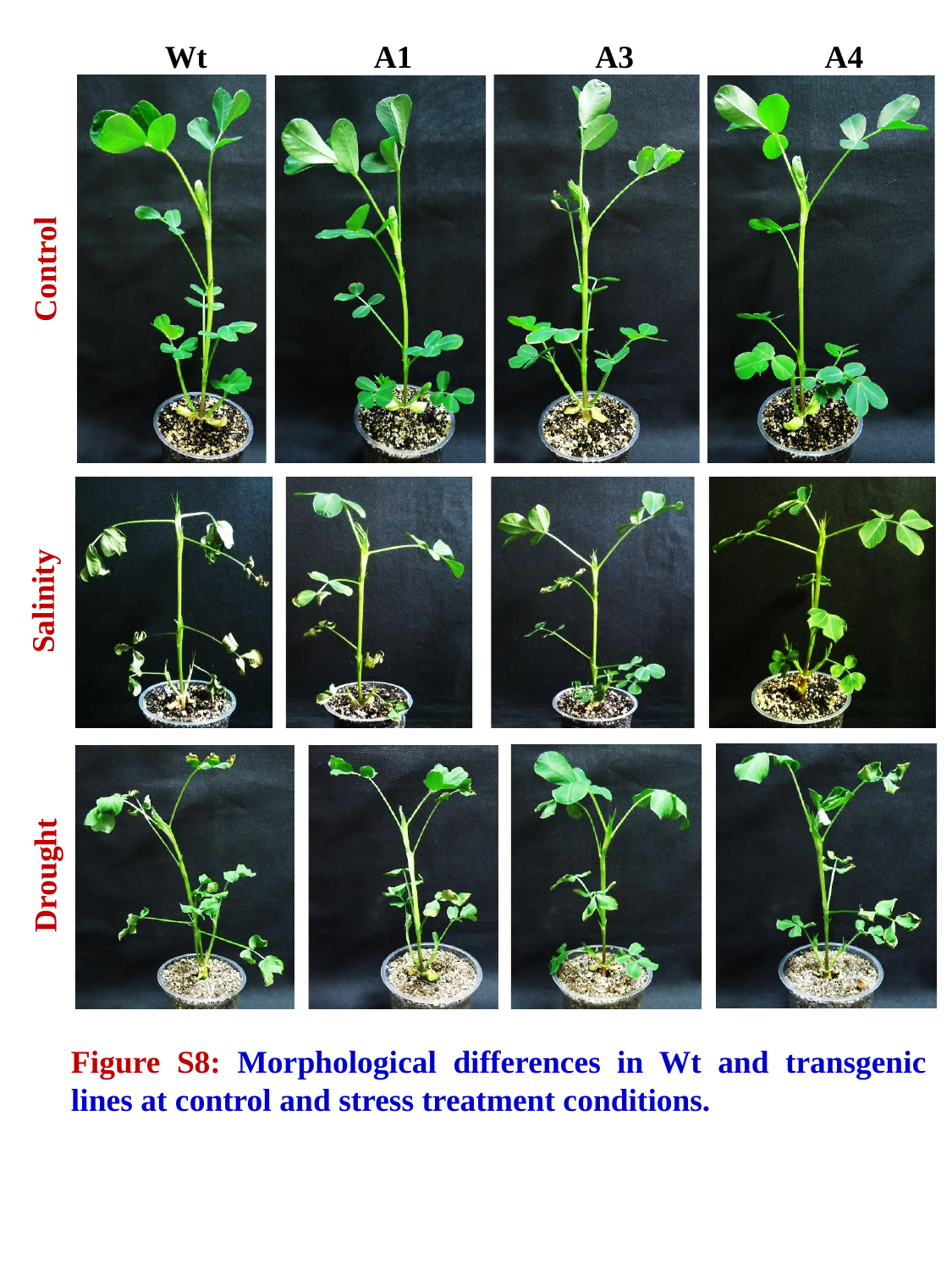

Wt A1 A3 A4
Control
Salinity
Drought
Figure S8: Morphological differences in Wt and transgenic lines at control and stress treatment conditions.
