# Supplementary material for: Introgression of the SbASR-1 Gene Cloned from a Halophyte Salicornia brachiata Enhances Salinity and Drought Endurance in Transgenic Groundnut (Arachis hypogaea) and Acts as a Transcription Factor
Source: PLoS One. 2015 Jul 9;10(7):e0131567. doi: 10.1371/journal.pone.0131567 (PMC4497679; doi:10.1371/journal.pone.0131567)
Supplement: S9 Fig — (PPTX) [file pone.0131567.s011.pptx]

## Slide 1
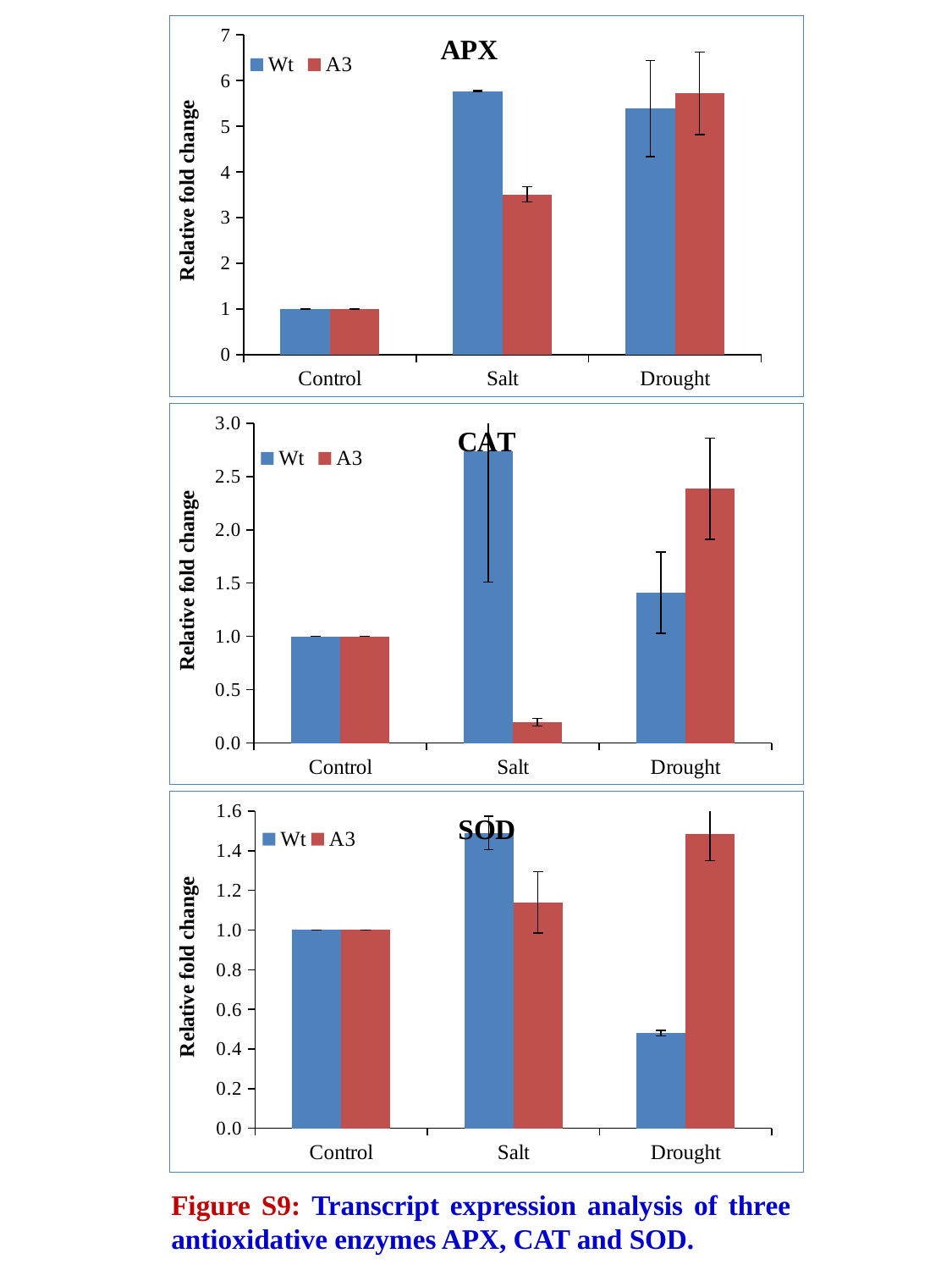

### Chart: APX
| Category | Wt | A3 |
|---|---|---|
| Control | 1.0 | 1.0 |
| Salt | 5.77 | 3.51 |
| Drought | 5.385 | 5.720000000000001 |
### Chart: CAT
| Category | Wt | A3 |
|---|---|---|
| Control | 1.0 | 1.0 |
| Salt | 2.74 | 0.195 |
| Drought | 1.41 | 2.385 |
### Chart: SOD
| Category | Wt | A3 |
|---|---|---|
| Control | 1.0 | 1.0 |
| Salt | 1.49 | 1.1400000000000001 |
| Drought | 0.48 | 1.4849999999999999 |Figure S9: Transcript expression analysis of three antioxidative enzymes APX, CAT and SOD.
