# Supplementary material for: Correction: Introgression of the SbASR-1 Gene Cloned from a Halophyte Salicornia brachiata Enhances Salinity and Drought Endurance in Transgenic Groundnut (Arachis hypogaea) and Acts as a Transcription Factor
Source: PLoS One. 2015 Aug 7;10(8):e0135541. doi: 10.1371/journal.pone.0135541 (PMC4529166; doi:10.1371/journal.pone.0135541)
Supplement: S1 File — (PDF) [file pone.0135541.s001.pdf]

PLOS ONE

RESEARCH ARTICLE

Introgression of the SbASR-1 Gene Cloned from a Halophyte *Salicornia braciata* Enhances Salinity and Drought Endurance in Transgenic Groundnut (*Arachis hypogaea*) and Acts as a Transcription Factor

Vivekanand Tiwari, Amit Kumar Chaturvedi, Avinash Mishra*, Bhavanath Jha*

Marine Biotechnology and Ecology Division, CSIR-Central Salt and Marine Chemicals Research Institute, Bhavnagar, Gujarat, India

* avinash@csmcri.org (AM); bjha@csmcri.org (BJ)

OPEN ACCESS

Citation: Tiwari V, Chaturvedi AK, Mishra A, Jha B (2015) Introgression of the SbASR-1 Gene Cloned from a Halophyte *Salicornia braciata* Enhances Salinity and Drought Endurance in Transgenic Groundnut (*Arachis hypogaea*) and Acts as a Transcription Factor. PLoS ONE 10(7): e0131567. doi:10.1371/journal.pone.0131567

Editor: Prabodh Kumar Trivedi, CSIR-National Botanical Research Institute, INDIA

Received: January 6, 2015

Accepted: June 3, 2015

Published: July 9, 2015

Copyright: © 2015 Tiwari et al. This is an open access article distributed under the terms of the Creative Commons Attribution License, which permits unrestricted use, distribution, and reproduction in any medium, provided the original author and source are credited.

Data Availability Statement: All relevant data are within the paper and its Supporting Information files.

Funding: This study was supported by the Council of Scientific and Industrial Research (CSIR, www.csir.res.in), Government of India, New Delhi (BSC0109–SIMPLE; BSC0117–PMSI, Senior Research Fellowship to AKC and VT). The funders had no role in study design, data collection and analysis, decision to publish, or preparation of the manuscript.

Abstract

The *SbASR-1* gene, cloned from a halophyte *Salicornia brachiata*, encodes a plant-specific hydrophilic and stress responsive protein. The genome of *S*. *brachiata* has two paralogs of the *SbASR-1* gene (2549 bp), which is comprised of a single intron of 1611 bp, the largest intron of the abscisic acid stress ripening [*ASR*] gene family yet reported. *In silico* analysis of the 843-bp putative promoter revealed the presence of ABA, biotic stress, dehydration, phytohormone, salinity, and sugar responsive *cis*-regulatory motifs. The *Sb*ASR-1 protein belongs to Group 7 LEA protein family with different amino acid composition compared to their glycophytic homologs. Bipartite Nuclear Localization Signal (NLS) was found on the C-terminal end of protein and localization study confirmed that *Sb*ASR-1 is a nuclear protein. Furthermore, transgenic groundnut (*Arachis hypogaea*) plants over-expressing the *SbASR-1* gene constitutively showed enhanced salinity and drought stress tolerance in the T1 generation. Leaves of transgenic lines exhibited higher chlorophyll and relative water contents and lower electrolyte leakage, malondialdehyde content, proline, sugars, and starch accumulation under stress treatments than wild-type (Wt) plants. Also, lower accumulation of H_2O_2 and $O_2^{\cdot-}$ radicals was detected in transgenic lines compared to Wt plants under stress conditions. Transcript expression of *APX* (ascorbate peroxidase) and *CAT* (catalase) genes were higher in Wt plants, whereas the *SOD* (superoxide dismutase) transcripts were higher in transgenic lines under stress. Electrophoretic mobility shift assay (EMSA) confirmed that the *Sb*ASR-1 protein binds at the consensus sequence (C/G/A)(G/T)CC(C/G)(C/G/A)(A/T). Based on results of the present study, it may be concluded that *Sb*ASR-1 enhances the salinity and drought stress tolerance in transgenic groundnut by functioning as a LEA (late embryogenesis abundant) protein and a transcription factor.

Competing Interests: The authors have declared that no competing interests exist.

Introduction

Abscisic acid stress ripening-1 (*ASR-1*) genes are stress and developmentally regulated plant-specific genes, first reported in tomato [1]. There are a number of paralogs of the *ASR* gene family in different plant groups [2]. The maize *ASR* gene family is the largest family found so far, and includes nine members, among which *ZmASR-1* is the most abundantly expressed gene [3]. *ASR-1* gene transcript expression has been reported to be induced by several abiotic and biotic factors [4]. The *Vv*MSA (grape ASR protein) is involved in the regulation of the cellular glucose level through ABA and the glucose signaling pathway via the *Vv*HXK1 and *Sn*RK1 proteins [5]. The role of the ASR-1 protein in the regulation of expression of sugar transporters and in the mobilization of sugar from the leaf to non-photosynthetic organs has been unveiled recently [4]. A separate group of LEA proteins—Group 7—was suggested exclusively for ASR proteins, based on conserved motifs present in the primary amino acid sequence of the protein [6]. The ASR-1 protein is found in an unstructured form in the cytosol and protects cytosolic proteins [7], whereas grape *Vv*MSA and *Sl*ASR-1 proteins were reported to function as a transcription factor when localized to the nucleus [8–9]. Also, a substantial antioxidant property was shown by *in vitro* studies of *Gm*ASR and *Os*ASR-1 proteins [10–11]. Overexpression of *ASR-1* genes in different plant species showed increased tolerance to oxidative, salinity and dehydration stress [12–14]. These reports suggest that *ASR-1* genes are potential candidates for genetic engineering of crops for better stress tolerance.

Halophytes are able to survive in the extreme environmental conditions due to their unique adaptation of tissue tolerance mechanisms. They complete their life cycle optimally at NaCl concentration of 200 mM in the soil and have ability to tolerate many fold higher concentration [15]. Halophytes, *Thellungiella halophila* and *Porteresia coarctata* displayed higher constitutive expression of several stress responsive genes and lower metabolic disturbances compared to their glycophytic relatives *Arabidopsis thaliana* and rice, respectively in a comparative proteomic analysis [16]. Ion transporters are more efficient in sequestration or extrusion of toxic ions than that of glycophytes. Moreover, toxic Na^+ and Cl^- ions are used as energetically cheap osmoticum to avoid diversion of extra energy to synthesize any osmolyte for maintaining the cellular osmotic balance with surrounding environments under moderately saline conditions [17]. Halophytes developed different composition of lipids, amino acid residues in proteins and free amino acids than that of glycophytes. The tonoplast of *Suaeda maritima* is abundant with highly saturated fatty acids and accumulated 30% cholesterol to avoid leakage of sequestered ions from vacuole to cytoplasm [17]. The negatively charged acidic amino acids glutamate and aspartate are most abundant in halophytes of Chenopodiaceae and Aizoaceae family. These amino acids are known to be accumulated in salinity stress conditions and act as osmoregulator [18].

Halophytes belong to half of the higher plant families, but contribute only 1% of the total plants species. The Chenopodiaceae or Amaranthaceae family constitutes highest number (total 281) of halophyte species and *Salicornia brachiata* Roxb (Amaranthaceae) is a leafless succulent annual halophyte that grows along coastal marshes. The plant has the capability to grow in a wide range of salt concentrations (0.1–2.0 M), accumulates salt up to 40% of its dry weight, and requires NaCl essentially for its *in vitro* regeneration [19]. Furthermore, the plants contain sulfur-rich seed storage proteins [20], unique oligosaccharides, and metabolites [21–22], and have proven to be a source of potential stress responsive genes for developing stress-tolerant transgenic plants [23–30]. Previously, the *SbASR-1* gene was cloned from *S. brachiata* and characterized by overexpressing in transgenic tobacco under salinity stress [12]. Groundnut or peanut (*Arachis hypogaea*) is an important cash crop with high nutritional value and many industrial uses. Groundnut is cultivated in the arid and semiarid region, and its

productivity is challenged by numerous abiotic stresses [31]. Among these abiotic stresses, salinity, drought, and heat are the major factors, limiting the productivity of groundnut [32]. High salinity adversely affects the groundnut productivity by reducing the seed germination, growth, dry matter synthesis, and mineral uptake [33]. Drought stress alone contributes to approximately 70% loss of peanut productivity worldwide [34]. It has been estimated that the annual loss in groundnut production due to drought stress was over US $520 million [35].

Genetic engineering approaches for crop improvement are comparatively faster than the classical breeding program, including cloning of genes responsible for important traits and introgression into crop plants to develop transgenics [36–40]. However, an efficient regeneration and genetic transformation protocol is a key step for the biotechnological program of any crop [41–43]. In recent years, there have been several reports on genetic engineering of groundnut for the improvement of tolerance against several stresses. Genetic transformation of groundnut with *AtAVP1*, *AtNHX1*, *PDH45*, and *SbpAPX* genes improved the biomass production, photosynthetic rates, and tolerance towards salinity and drought stress [28, 44–46]. An improved yield potential was observed in transgenic groundnut overexpressing the *AtNAC2* gene compared to wild-type (Wt) plants under drought and salinity stress conditions [47]. Similarly, it has been reported that overexpression of the *IPT* gene under the control of a maturation- and stress-inducible promoter, improves the drought tolerance, and produces 51–65% higher yield of groundnut in field conditions without affecting the oil quality [48].

In the present study, the *cis*-regulatory motifs were identified in the promoter region of the *SbASR-1* gene. The genomic organization of the *SbASR-1* gene and the copy number were determined. A local groundnut cultivar GG-20 was transformed with the *SbASR-1* gene to enhance the salinity and drought stress tolerance of the plant. This study is the first report on the *cis*-regulation, genomic organization, and copy number of a halophytic *ASR-1* gene and the functional characterization in a crop plant. Analysis of the T1 transgenic lines confirmed the enhanced tolerance of plants against salinity and drought stress.

Materials and Methods

Isolation of the *SbASR-1* promoter region and *in silico* analysis

The gene-specific primers (**S1 Table**) were designed from the cDNA sequence of the *SbASR-1* gene (accession no.: EU746399), and the promoter region was isolated using the genome-walking technique [25]. The putative promoter was cloned and sequenced (accession no.: KM462537). *In silico* analysis was performed for the presence of *cis*-regulatory elements using the online program PLACE [49].

Disorder promoting amino acids were identified [7] and their percent composition in *Sb*ASR-1 protein was determined by BioEdit software (ver. 7.0.7.1). The N-myristoylation sites and characteristic conserved domain of ASR family were predicted through online server PROSCAN (PROSITE SCAN) and InterPro (http://www.ebi.ac.uk/Tools/pfa/iprscan/), respectively.

Genomic organization and copy number of *SbASR-1*

The *SbASR-1* gene was amplified using genomic DNA (100 ng) as the template and the primer pair ASRP-ASRR (**S1 Table**). The amplicons were cloned, sequenced, and submitted to NCBI (accession no.: KM462537). The exon and intron regions were detected by comparing the genomic sequence of the gene with the cDNA sequence (EU746399). The copy number of the *SbASR* gene was determined by Southern hybridization. About 25 μg of genomic DNA was digested with *Eco*RI, *Hind*III, or *Xba*I, then separated on a 0.7% (w/v) agarose gel, and blotted onto a Hybond (N^+) membrane (GE Healthcare Life Sciences, Pittsburgh, Pennsylvania, USA).

Hybridization was performed with an ASR gene probe of 368 bp, which was prepared from the cDNA sequence of *SbASR-1* using the PCR DIG Probe Synthesis kit (Roche, Basel, Switzerland). The detection of hybridized DNA was performed using the CDP-Star detection kit (Roche, Switzerland). The developed X-ray films were scanned with a Densitometer (model GS-800, Bio-Rad, Hercules, California, USA).

Heterologous expression of *Sb*ASR-1 protein

The coding sequence of the *SbASR-1* gene was cloned into the pET28a expression vector using the primer pair PARF-PARR (**S1 Table**), and was confirmed by sequencing. The cloned plasmid was transformed into *E. coli* BL21 (DE3) cells. Recombinant protein expression was induced by adding 1 mM isopropyl *β*-D-1-thiogalactopyranoside (IPTG) when the culture turbidity reached 0.6 OD at 600 nm. The optimal time required for a sufficient level of protein expression after induction was determined on sodium dodecyl sulfate-polyacrylamide gel electrophoresis (SDS-PAGE). The *Sb*ASR-1 protein was purified using QIA*express* Ni-NTA Fast Start kit (Qiagen, Hilden, Germany) following manufacturer's protocol. The purity and size of the protein was evaluated by 12.5% SDS-PAGE.

Subcellular localization of a *Sb*ASR-1:RFP translational fusion protein

The full length *SbASR-1* cDNA was amplified with gene specific primers ASRLoF-ASRLoR (**S1 Table**) using *Pfx* DNA polymerase. The blunt end PCR product (*SbASR-1* gene) was cloned into *attL*-containing pENTER/D-TOPO entry vector (Invitrogen, USA) and confirmed by sequencing. The recombinant vector *pENTER*:*SbASR-1* was subjected for the LR recombination with *attR*-containing destination vector pSITE-4CA using LR Clonase II enzyme mix (Invitrogen, USA). Reading frame of fusion gene construct (*RFP*:*SbASR-1*) was confirmed by sequencing, thereafter transformed into onion epidermal cells by particle bombardment (PDS-1000/He Biolistic, Biorad, USA). The pSITE-4CA vector containing RFP was used as control. Transformed onion epidermal cells were stained with DAPI (for nuclear staining) after incubating for 24 h on MS medium. Sub-cellular localization analysis was performed by observing the site of transient expression of RFP under different filters using an epifluorescence microscope (Axio Imager, Carl Zeiss AG, Germany).

*Sb*ASR-1 protein binding with DNA probe

The DNA-binding property of the *Sb*ASR-1 protein was studied using the electrophoretic mobility shift assay (EMSA). Eight DNA probes (ARBS-1 to ARBS-8; **S1 Fig**) were designed from small fragments of DNA probes, as used by Rom *et al.* [50] and and Ricardi *et al.* [9], with a modified consensus binding site. ARBS-1 to ARBS-4 had consensus binding sites as reported by Rom *et al.* [50], whereas the remaining four probes (ARBS-5 to ARBS-8) had consensus binding sites as suggested by Ricardi *et al.* [9]. The probes were prepared by 3'-end-labeling using the DIG-11-ddUTP labeling kit (Roche, Switzerland). The binding was performed at room temperature for 30 min in HEPES-KOH buffer (20 mM HEPES-KOH, pH 7.6), 30 mM KCl, 1% (v/v) Tween-20, 10 mM $(NH_4)_2SO_4$, 0.01 mg/ml BSA, 10% glycerol, and 5 mM $ZnCl_2$). The DNA/protein complexes were electrophoresed on 5% non-denaturing polyacrylamide gels at 100 V for 1.5 h at 4°C. The running buffer (0.5X TBE buffer) also contained the same concentration of glycerol and BSA. The complex was transferred onto Nylon Hybond$^+$ membrane by electroblotting and detected using the CDP-Star detection kit (Roche, Switzerland).

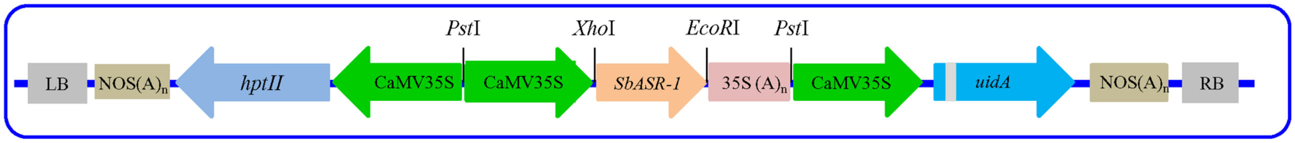

Fig 1. Schematic representation of *SbASR-1-pCAMBIA1301* plant transformation vector construct. The *SbASR-1* gene expression is regulated by CaMV35S promoter and terminator. A *gus* gene is used as reporter while *hptII* as selectable marker in T-DNA region of construct.

doi:10.1371/journal.pone.0131567.g001

Genetic transformation and regeneration of transgenic groundnut plants

The *SbASR-1* gene was genetically transformed to the groundnut using *SbASR-1:pCAMBIA1301* plant expression gene construct (Fig 1). De-embryonated cotyledon explants were prepared and transformed in different batches (each batch containing approximately 1000 explants) using the *Agrobacterium*-mediated transformation method [51]. Transformation efficiency was calculated by histochemical GUS assay. Regenerated putative transformed shoot buds were elongated and grafted onto non-transformed stocks [51]. After 3 weeks, new leaves emerged, and plants were acclimatized and transferred to a greenhouse for further growth and seed development.

Confirmation of the transgene integration

The transgene integration into the groundnut genome was confirmed by polymerase chain reaction (PCR) and Southern hybridization. Total genomic DNA from the hardened T0 transgenic lines and Wt plants were isolated from expanded leaves. The PCR reactions were set to screen for the presence of the *SbASR-1* gene using specific primer pairs ASRF-ASRR (S1 Table). Each PCR reaction was performed in 25 μl of the reaction volume with 1x reaction buffer supplemented with 1.5 mM $MgCl_2$, 0.2 mM dNTPs, 5 pmol of each primer, 1.25 units of *Taq* DNA polymerase, and 200 ng of plant genomic DNA. The amplicons were electrophoresed on a 1.0% agarose gel, detected by ethidium bromide, and photographed using a Gel Doc system (Bio-Rad, USA).

For Southern blot hybridization analysis, about 10 μg of genomic DNA was digested with *Hind*III. Digested DNA fragments were blotted and hybridized with DIG-11-dUTP-labeled *ASR-1*-specific DNA probes, which was synthesized using gene specific primers ASRF-ASRR (S1 Table). Blastn was performed for the probe sequence to check any similarity with the endogenous *ASR-1* gene sequence of *Arachis hypogaea*. The hybridized membrane was developed using CDP-Star as the substrate (Roche, Switzerland), and signals were visualized on X-ray film. The developed X-ray films were scanned with a Densitometer (Bio-Rad, USA).

Transgene expression analysis in the transgenic lines

Total RNA was isolated from leaf tissues of transgenic lines and Wt plants using the RNeasy Plant Mini Kit (Qiagen, Germany). The cDNA were prepared using reverse transcriptase (ImProm-II Reverse Transcriptase, Promega, Madison, Wisconsin, USA) and used for PCR containing 100 ng cDNA, 10 pmol of *ASR-1* primers ASRF–ASRR or *Ah-actin* primers AhACTF–AhACTR (S1 Table), 200 μM dNTPs, and 2.5 U *Taq* DNA polymerase in a 50-μl reaction. The PCR products were analyzed on a 1.5% (w/v) agarose gel.

Transient GUS expression in de-embryonated cotyledon explants (just after co-cultivation) and stable expression in leaves were assessed by using *β*-Glucuronidase Reporter Gene Staining Kit (Sigma, St. Louis, Missouri, USA). Transformed explants after co-cultivation and leaves of hardened putative transgenic plants were washed with water, blotted, and dipped into the

staining buffer overnight at 37°C in the dark. The tissues were de-stained using 70% (v/v) ethanol, and leaves were photographed.

Analysis of transgenic plants under stress conditions

Seeds of transgenic lines and Wt plants were surface sterilized, germinated on wet filter papers for 3 days in the dark, and then transferred to Soilrite (Keltech Energy Ltd., Bengaluru, India). Leaves from all five T1 transgenic lines and Wt plants were screened for GUS expression, and three lines that gave the best expression were selected for further analysis.

Leaf discs of Wt plants and three transgenic lines (A1, A3, and A4) of the T1 generation were immersed in ½ MS (Murashige and Skoog) salt solution supplemented with or without 250 mM NaCl or 15% (w/v) PEG-6000 in a 16/8-hour light/dark photoperiod for 1 week in a culture room. Seedlings (7 days old) were used for salinity and drought stress treatments. For salinity stress, 250 mM NaCl solution in water was used for irrigation every other day (for 2 weeks), whereas for drought stress, irrigation was stopped for 2 weeks.

Leaf senescence and estimation of photosynthetic pigments

Leaf discs of 5-mm diameter were excised from detached leaves, and six discs of each line in duplicate were used in a 24-well culture plate. The leaf discs were kept under a photoperiod of 16-h white lights (35μmol m^{-2} s^{-1}) and 8-h dark at 25°C ± 2°C for 7 days. The fresh weight of air-dried leaf discs was recorded, and then the discs were homogenized in 2 ml of chilled *N*,*N*-dimethylformamide (DMF) on ice in the dark. The homogenates were spun at 3000 g for 10 min, and quantitative estimation of chlorophylls (Chl a, Chl b, and total Chl) and carotenoids was carried out [52–53].

Relative water content, lipid peroxidation, and electrolyte leakage

Relative water content (RWC) and electrolyte leakages (EL) were measured using leaf discs (2 cm^2) of transgenic lines and Wt plants [12, 54]. Lipid peroxidation was studied by estimating the total malondialdehyde (MDA) content [55].

Estimation of free proline, and total soluble sugar, reducing sugar, and starch

Free proline contents were estimated using 100 mg of leaf tissue of transgenic lines and Wt plants. Tissues were homogenized in 3% (w/v) sulfosalicylic acid, spun at 10,000 g for 10 min at 4°C, and the proline content was estimated using ninhydrin reagent [56]. Total soluble sugars and starch contents were estimated using anthrone reagent [57], while reducing sugar was estimated by the DNS method [58].

In vivo localization of peroxide and superoxide radicals

Superoxide radicals were detected by immersing leaf samples in NBT solution (1 mg/ml in 10 mM phosphate buffer; pH 7.8), whereas for *in vitro* localization of H_2O_2, leaves were incubated in DAB solution (1 mg/ml in 10 mM phosphate buffer; pH 3.8) at room temperature for 2 h and 6 h, respectively in the dark. Thereafter, samples were exposed to light for the appearance of spots. Pigments were bleached by treating leaf samples with destaining solution (ethanol: acetic acid: glycerol; 3:1:1 v/v) for 15 min at 95°C, and then samples were documented.

Transcript expression analysis of genes encoding antioxidative enzymes

Total RNA of Wt plant and transgenic line A3 was isolated, and cDNAs were prepared using reverse transcriptase. Changes in the transcript expression of *APX* (ascorbate peroxidase), *CAT* (catalase), and *SOD* (superoxide dismutase) genes were studied by quantitative PCR (qPCR) using the QuantiFast SYBR Green PCR reaction kit (Qiagen, Germany). The reaction mixture included 100 ng of cDNA, 0.16 μM of primers in 25 μl of 1X QuantiFast SYBR Green PCR mix. Reactions were run in a Real-Time iQ5 Cycler (Bio-Rad, USA). Along with previously reported primers for *APX* and *SOD* [59], new primers were designed for *CAT* (**S1 Table**). At the end of the qPCR cycles, the products were put through a melting-curve analysis to determine the specificity of amplification. The fold changes of transcripts were determined by the comparative $2^{-\Delta\Delta Ct}$ method [60], and stress-treated plants (transgenic and Wt) were compared with controls (unstressed plants). The *Ah-actin* gene was used as an internal control to normalize the qPCR reactions.

Statistical analyses

All the experiments were carried out twice with three biological replicates. Analysis of variance (ANOVA) was performed, and significance was determined at $P \leq 0.05$. Mean values that were significantly different within a treatment from each other are indicated by different letters. The standard deviation (SD) was calculated to show the variation in the replicates, and bars represent the mean values ± SD.

Results

The *SbASR-1* gene promoter

The 949-bp upstream region of the *SbASR-1* gene was isolated from *S. brachiata* genomic DNA using the genome-walking method. After alignment with the gene sequence, a fragment of 843 bp upstream from the putative transcription start site (TSS) was identified as the promoter region of the *SbASR-1* gene (**Fig 2**). *In silico* analysis of the promoter sequences identified a TATA box at 32 bp upstream to the putative TSS, which further confirmed the TSS site. The putative *cis*-regulatory motifs of the gene were categorized into seven different categories: ABA and dehydration responsive; light responsive; metal responsive; phytohormone responsive; pollen and embryo specific; tissue/organelles specific; and pathogen responsive (**Table 1**). One identified motif, ABRELATERD1 (ACGTG), is involved in ABA-responsive expression of genes under abiotic stress conditions. The early response to dehydration (erd) motif, ACGTATERD1 (ACGT), was identified at three sites on both strands. The MYB1AT motif, known for the binding of MYB2 and MYC2 transcription factors, was identified at three sites on the plus strand and two sites on the complementary strand. Core binding sites for MYB1 and MYB2 (MYBCORE) were detected at four sites (three on the plus strand, and one on the complementary strand). Four MYB2CONSENSUSAT motifs were also identified in the promoter region. A pathogen- and salt stress-inducible GT-1 motif was identified on the complementary strand. A number of light-responsive motifs, including CIACADIANLELHC (3 repeats), EBOXBNNAPA (8 repeats), GATABOX (8 repeats), GT1CONSENSUS (6 repeats), GT1CORE (2 repeats), IBOXCORE (3 repeats), PRECONSCRHSP70A (1 repeat), SORLIP2AT (8 repeats), and TBOXATGAPB (2 repeats), were found on both plus and complementary strands. Two copper-responsive motifs (CURECORECR) and one zinc-inducible motif WBBOXPCWRKY1 were also detected. Numerous embryo-specific (CANBNNAPA, SEF4MOTIFGM7S) and pollen-specific (GTGANTG10, POLLEN1LELAT52) motifs were found in the putative promoter

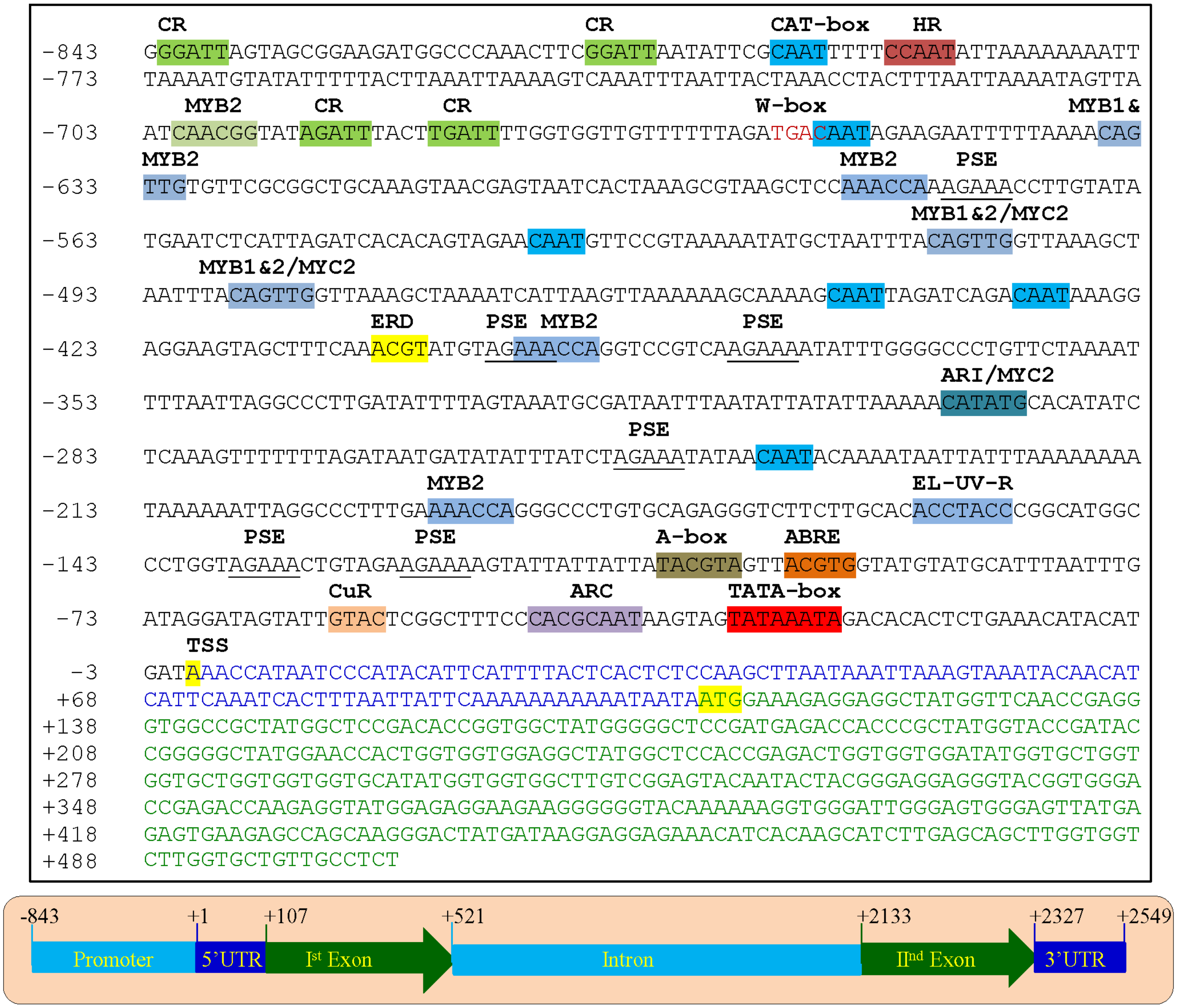

Fig 2. Graphical representation of *SbASR-1* gene genomic organization. Total length of the gene is 2549 bp, which comprised of 106 bp 5'-UTR, 414 bp exon I, 1611 bp intron, 195 bp second exon II and 224 bp 3'-UTR. Upstream promoter region of 843 bp contains TATA-box at -32 bp position.

doi:10.1371/journal.pone.0131567.g002

region (Table 1). It seems that, apart from ABA, other phytohormones also play a crucial role in the regulation of expression of the *SbASR-1* gene. Motifs inducible to phytohormones, such as auxin, cytokinin, gibberellic acid (GA), and salicylic acid (SA), were found in abundance in the *SbASR-1* gene promoter (Table 1). Two A-box motifs were identified, one on each strand. The A-box motif is reported to be involved in sugar metabolism of plants, specifically during sugar repression.

Genomic organization and copy number of the *SbASR-1* gene

The *SbASR-1* gene (2549 bp) was comprised of a 5'-UTR (106 bp), and an intron of 1611 bp that was flanked by exon I (414 bp) and exon II (195 bp), followed by a 223-bp 3'-UTR region (Fig 2). In Southern blotting, *Eco*RI-digested DNA gave two intense bands and two light bands (shown in S2 Fig with arrows). Similarly, four intense bands were detected with *Xba*I-digested

Table 1. Different *cis*-regulatory motifs identified on *SbASR-1* promoter by PLACE online program.

Category	Motif	Consensus Sequence	Function	Number of motifs
ABA and dehydration responsive	ABRELATERD1	ACGTG	ABA-responsive element	1 (+)
	ACGTATERD1	ACGT	Etiolation-induced early responsive to dehydration	3 (+), 3 (-)
	BOXLCOREDCPAL	ACCWWCC	Elicitor and UV induced MYB1 binding site	1 (+)
	CCAATBOX1	CCAAT	Heat responsive	1 (+)
	GT1GMSCAM4	GAAAAA	Pathogen and salt inducible GT-1 motif	1 (-)
	MYB1AT	WAACCA	Drought responsibe MYB2 and MYC2 binding site	3 (+), 2 (-)
	MYB2CONSENSUSAT	YAACKG	Drought responsive MYB2 and MYC2 binding consensus sequence	1 (+), 3 (-)
	MYBCORE	CNGTTR	MYB1 and MYB2 protein involved in dehydration and flavonoid biosynthetic pathway responsive	3 (+), 1 (-)
	MYCCONSENSUSAT	CANNTG	Binding site of AtMYC2	4 (+), 4 (-)
Light responsive	CIACADIANLELHC	CAANNNNATC	Circardian expression, light responsive	1 (+), 2 (-)
	EBOXBNNAPA	CANNTG	Light and tissue specific expression	4 (+), 4 (-)
	GATABOX	GATA	Light and tissue specific expression	6 (+), 2 (-)
	GT1CONSENSUS	GRWAAW	Light responsive	3 (+), 3 (-)
	GT1CORE	GGTTAA	Light responsive	2 (+)
	IBOXCORE	GATAA	Light responsive	2 (+), 1 (-)
	PRECONSCRHSP70A	SCGAYNR(N) 15HD	Plastid response element, light and MgProto inducible	1 (+)
	SORLIP2AT	GGGCC	Light and phytochromeA inducible gene expression	2 (+), 6 (-)
	TBOXATGAPB	ACTTTG	T-BOX, light inducible	2 (-)
Metal responsive	CURECORECR	GTAC	Copper responsive elements	1 (+), 1 (-)
	WBBOXPCWRKY1	TTTGACY	Elicitor and zinc dependent WRKY binding sites	1 (-)
Phytohormone responsive	ARR1AT	NGATT	Binding site of cytokinin regulated transcription factor ARR1	4 (+), 4 (-)
	ASF1MOTIFCAMV	TGACG	binding site of ASF-1, transcriptional activation in auxin and salicyalic acid responses	1 (-)
	CACGCAATGMGH3	CACGCAAT	Auxin responsive, constitutive expression	1 (+)
	CATATGGMSAUR	CATATG	Auxin inducible expression	1 (+), 1 (-)
	WBOXATNPR1	TTGAC	W-box, Salicyalic acid inducible binding of WRKY	2 (-)
	WRKY71OS	TGAC	W-box core sequence, binding site of WRKY71, transcriptional repressor of gibberellin signalling pathway	1 (+), 2 (-)
Pollen and embryos specific	CANBNNAPA	CNAACAC	Embryo and endosperm specific expression	1 (-)
	GTGANTG10	GTGA	Late pollen gene specific GTGA motif	2 (-)
	POLLEN1LELAT52	AGAAA	Pollen specific expression	6 (+)
	SEF4MOTIFGM7S	RTTTTTR	Soybean embryo factor (SEF-4) binding site	2 (+), 2 (-)
Tissue/ organelles specific expression	BOXIINTPATPB	ATAGAA	Box II elements found in few plastid gene promoters	1 (+)
	CACTFPPCA1	YACT	Mesophyll specific expression in C4 plants	7 (+), 10 (-)
	L1BOXATPDF1	TAAATGYA	HDZip and epidermal layer specific MYB binding site	1 (-)
	TAAAGSTKST1	TAAAG	Guard cell specific gene expression of Dof1	4 (+), 1(-)
Sugar responsive	ACGTABOX	TACGTA	A-Box, responsible for sugar repression	1 (+), 1 (-)
Pathogen responsive	BIHD1OS	TGTCA	binding site for bell homeodomain transcription factor, in diesease resistance response	1 (-)

doi:10.1371/journal.pone.0131567.t001

DNA; however, the *Hind*III-digested DNA sample gave only two bands (S2 Fig). Thus, Southern analysis revealed the presence of at least two paralogs of *SbASR-1* gene.

In silico analysis of *Sb*ASR-1 protein sequence

A special Group 7 of LEA protein was proposed exclusively for ASR-1 proteins based on conserved motifs. Out of five conserved motifs (m-1 to m-5) described for this group, the *Sb*ASR-1 contains four motifs, m-1 to m-3, and m-5 arranged in an order of 3-1-2-5 (S3 Fig). However, only first three motifs (1, 2, and 3) are essential for membership of this group. Presence of these motifs suggests that *Sb*ASR-1 is a Group 7 LEA protein. Comparative *in silico* analysis of the primary amino acid sequence of *Sb*ASR-1 and other selected ASR-1 proteins exhibited variation in amino acid composition of *Sb*ASR-1. The *Sb*ASR-1 protein has higher percent composition of glycine residue (25.25%), percent of disorder promoting amino acid residues (66.3%) and predicted N-myrystolation sites (29 sites) than that of other glycophyte ASR-1 proteins (Table 2). However *Sb*ASR-1 protein did not show much variation with another halophytic *Sl*ASR-1 (*Suaeda liaotungensis*) protein.

Table 2. Comparative analysis of *Sb*ASR-1 protein primary amino acid sequence with its homologs.

ASR-1	Protein accession	No. of amino acids	Disorder promoting amino acids (%)	Glycine content (%)	N-terminal His-rich domain	N-Myrostylationsite
Salicornia brachiata	ACI15208	202	66.3	25.25	No	29
Suaeda liaotungensis	AGZ20206	237	70.89	24.47	No	26
Oryza sativa	AAB96681	138	49.2	9.42	Yes	2
Citrus maxima	AAA82741	98	64.28	11.22	No	4
Solanum lycopersicum	AAY97998	110	59.09	8.18	Yes	1
Vitis vinifera	AAZ93634	149	54.36	6.04	Yes	1
Solanum lycopersicum	AAB64185	115	58.26	6.96	Yes	1
Lilium longiflorum	AAM51877	142	54.93	7.75	Yes	2
Solanum tuberosum	AAD00254	109	59.63	8.26	Yes	1
Cucumis melo	AAL27560	112	58.03	7.14	Yes	1
Ricinus communis	XP_002524296	141	58.86	5.67	Yes	1
Solanum cheesmaniae	AAY97997	111	58.56	8.11	Yes	1
Solanum lycopersicum	AAA34137	115	58.26	6.96	Yes	1
Solanum chilense	AAY98001	110	59.01	8.18	Yes	1
Solanum corneliomuelleri	AAY98002	110	59.09	8.18	Yes	1
Lycopersicon peruvianum	AAY98000	110	59.09	8.18	Yes	1
Solanum habrochaites	AAY97999	110	59.09	8.18	Yes	1
Ginkgo biloba	AAR23420	181	55.25	12.71	Yes	7
Vitis pseudoreticulata	ABC86744	149	54.36	6.71	Yes	1
Musa acuminata	ACL68147	146	60.27	6.85	Yes	2
Hevea brasiliensis	AAP46155	108	61.11	9.26	Yes	1
Musa acuminata	ACZ60128	143	58.74	6.99	Yes	1
Prunus armeniaca	AAB97140	200	64.5	21.5	Yes	1
Prunus mume	BAI94530	205	64.39	22.44	Yes	14
Prunus persica	AAL26889	193	63.73	19.17	Yes	10

doi:10.1371/journal.pone.0131567.t002

Heterologous expression of the *Sb*ASR-1 protein

*Sb*ASR-1 protein expression in the *E. coli* BL21 strain was induced by addition of 1 mM IPTG to the bacterial culture at the log-phase stage. The optimum time required for an efficient level of expression was found to be 6 h after induction (S4 Fig). Following optimization, protein was expressed in a 250-ml culture, and was purified using a Ni-NTA column (Qiagen, Germany). The purified protein had a size of approximately 32–34 kDa (S5 Fig), which is greater than the theoretical molecular mass of the *Sb*ASR-1 protein (21.01 kDa). However, elute 1 and elute 2 (E1 and E2) had several non-specific bands, which subsequently disappeared in E3 to E5.

Subcellular localization of RFP:*Sb*ASR-1 fusion protein

In silico analysis of *Sb*ASR-1 protein sequence showed the presence of a putative bipartite nuclear localization signal at C-terminal region. Furthermore, the subcellular localization study, performed with *RFP*:*SbASR-1* fusion construct confirmed that *Sb*ASR-1 is a nuclear protein (Fig 3). Transient RFP:*Sb*ASR-1 expression was aligned with nucleus of transformed onion epidermal cells. In contrast, evenly distributed red fluorescence signals were observed in the entire cell region of the onion cell transformed with RFP alone.

Binding of *Sb*ASR-1 to DNA

All eight DNA probes (ARBS-1 to ARBS-8) used in DNA/protein interaction screening study (S1 Fig), showed clear shifting of band (S6 Fig). Further, EMSA was reported with a probe ARBS-8 in the presence of non-labeled specific competitors, which confirmed the specificity of the band shifting, appeared due to binding of *Sb*ASR-1 with probe (Fig 4). Binding of *Sb*ASR-1

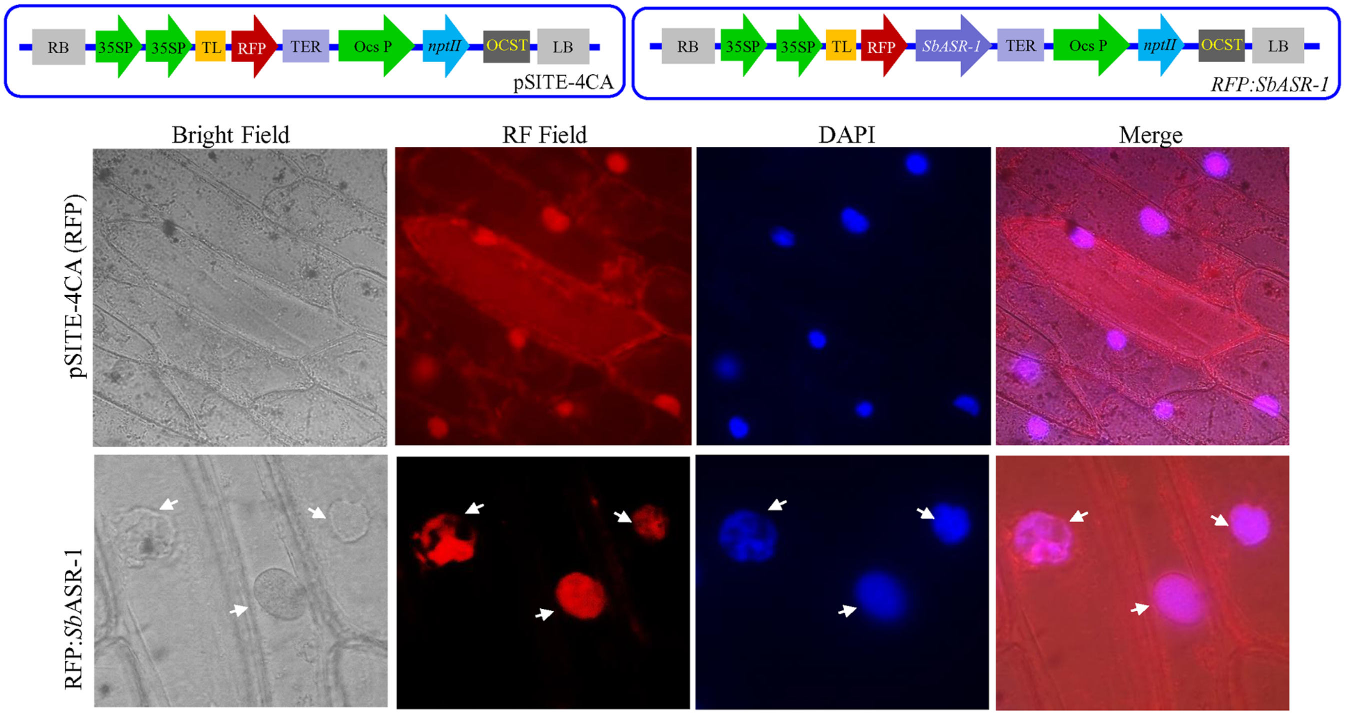

Fig 3. Subcellular localization of *Sb*ASR-1:RFP translational fusion protein in onion epidermal cells. Upper panel shows schematic map of pSITE-4CA (RFP) vector and *SbASR-1*:*RFP* gene construct. Lower panel shows transient expression of vector alone and recombinant gene construct on transformed onion epidermal cells analyzed under bright and red fluorescence field.

doi:10.1371/journal.pone.0131567.g003

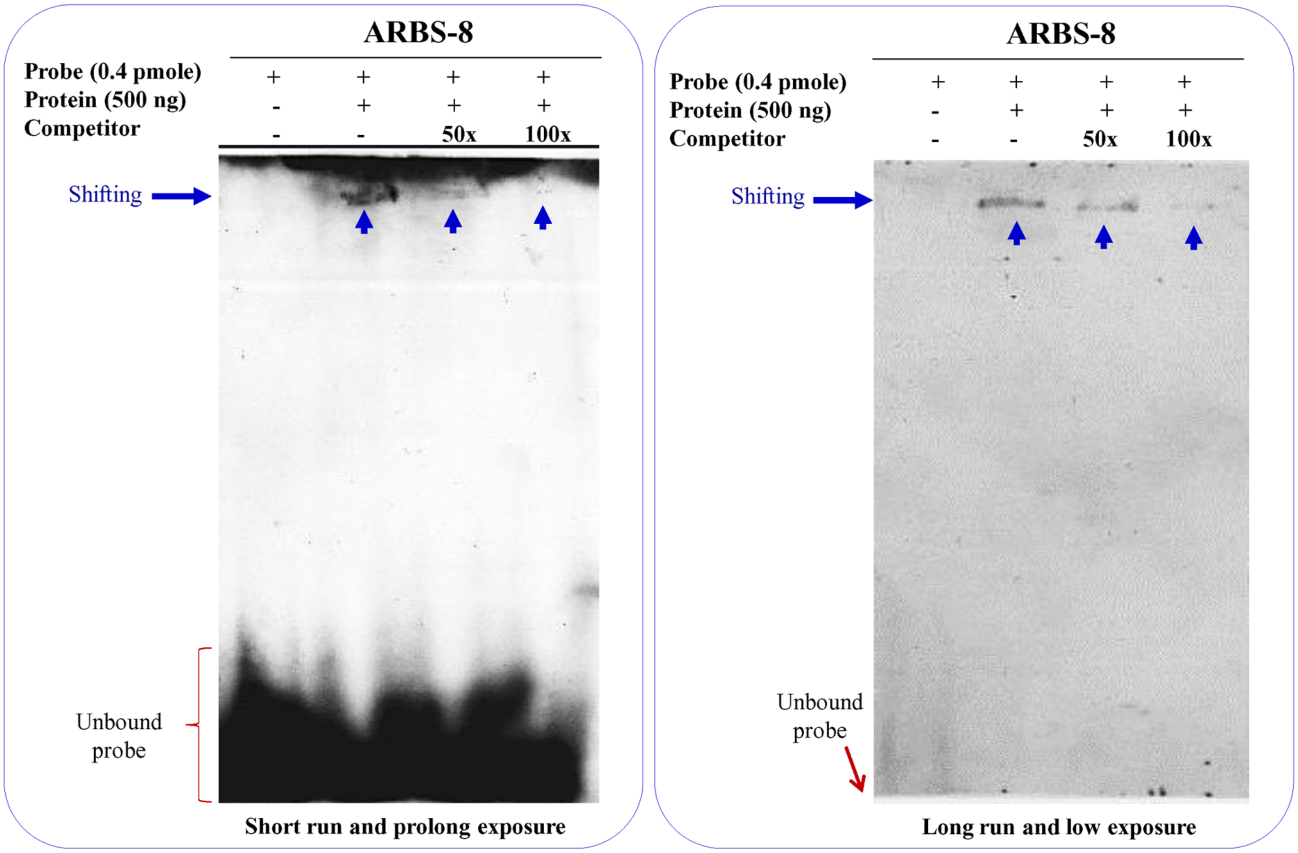

Fig 4. Binding of heterologously expressed recombinant *Sb*ASR-1 protein with DNA. EMSA was performed with or without presence of specific competitor. As the molar concentration of specific competitor (unlabelled ARBS-8) increased, the intensity of band decreased accordingly. It confirms the specificity of protein binding.

doi:10.1371/journal.pone.0131567.g004

with the ARBS-8 probe suggests that *Sb*ASR-1 acts as a transcription factor and binds with a consensus (C/G/A)(G/T)CC(C/G)(C/G/A)(A/T) sequence (Fig 4; S1 and S6 **Figs**).

Genetic transformation and regeneration of transgenic plants

After transformation, explants were regenerated (14.65±1.06% efficiency) on SM-3 media (Fig 5A–5C). After three rounds of selection on SLM-3 media, 3.82±0.6% explants survived (Fig 5D and 5E), whereas non-transformed explants died after the 2nd sub-culturing. Finally, an average of 37.92±1.5% hygromycin-resistant shoots was obtained relative to regenerated explants. Elongated shoots (Fig 5F) of 2–3 cm were then grafted onto 1-week-old non-transformed stocks, and after 21 days, new leaves emerged from the grafted scions (Fig 5G–5J). Putative transgenic plants were acclimatized under laboratory conditions and subjected to molecular confirmation. Positive transgenic lines were transferred to a greenhouse for further growth, and seeds were harvested after maturation (Fig 5K–5Q).

Molecular confirmation and transgene overexpression

Acclimatized putative transgenic lines were confirmed for the stable integration of the transgene into the groundnut genome by PCR and Southern hybridization. Amplification of the partial length of the *SbASR-1*gene (368 bp) was observed in all five transgenic lines and the positive control, while amplification was not detected in Wt plants (S7 Fig).

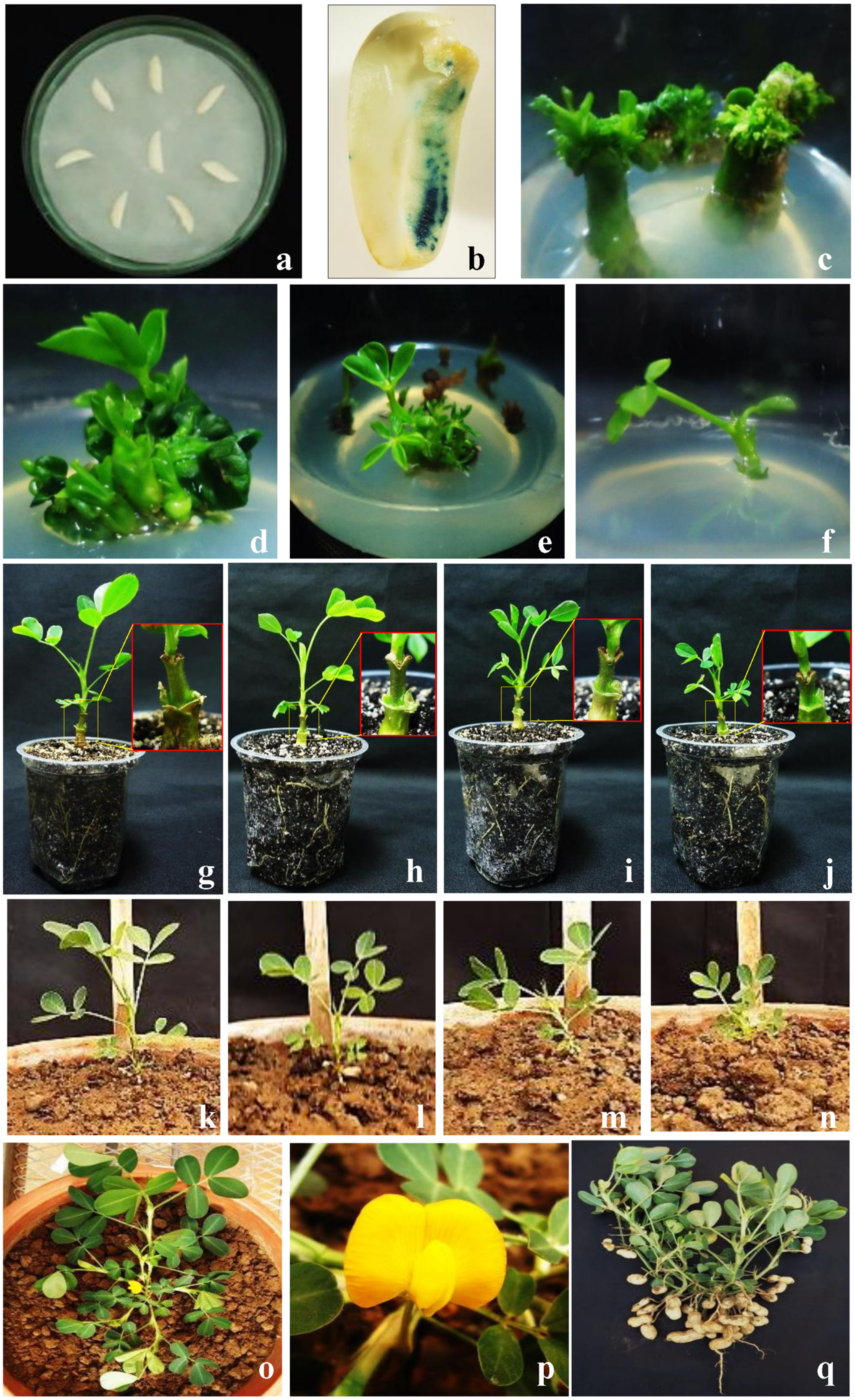
a
b
c
d
e
f
g
h
i
j
k
l
m
n
o
p
q

Fig 5. Genetic transformation and tissue culture of putative transgenic lines. Co-cultivation of deembryonated cotyledon explants (a), transient GUS expression (b), regeneration, selection, elongation, rooting and grafting of putative transgenic shoots (c-j), hardening (k-n), flowering (o-p) and seed harvesting (q).

doi:10.1371/journal.pone.0131567.g005

Stable integration of the transgene into the genome of all five transgenic lines was confirmed by Southern hybridization using a *SbASR-1* gene-specific probe (S7 Fig). All five transgenic lines showed a band of more than 4.36 kb, confirming single-copy integration of T-DNA into the groundnut genome (S7 Fig). Line A1 and A2 showed a hybridization signal at same position. Similarly, lines A4 and A5 also showed bands at the same position (as each other). The DNA from the Wt plants did not show any hybridization. Blastn analysis of *SbASR-1* probe sequence used in Southern hybridization experiment did not show any similarity with the *Arachis hypogaea* (S2 Table). It confirmed that the bands appeared in the Southern hybridization experiment is due to hybridization of probe with the transgene integrated into the genome.

Normal expression of the transgene was confirmed by reverse transcriptase PCR (RT-PCR) in transgenic lines at the transcript level (Fig 6A), and at the protein level by GUS histochemical assay (Fig 6B). PCR amplification of the *SbASR-1* gene using cDNA as a template amplified a 368-bp product in all transgenic lines, but there was no such product in Wt plants. Stringency of the reaction was checked by amplifying the *Ah-actin* gene using the same cDNA and PCR conditions. An amplicon of 174 bp was obtained in all transgenic lines and the Wt plants (Fig 6A). RT-PCR further confirms the stable integration and expression of the transgene.

Analysis of T1 transgenic lines

Seeds from the T0 transgenic lines were harvested from the greenhouse, sterilized, and germinated under laboratory conditions along with the Wt seeds. The transgenic lines were screened

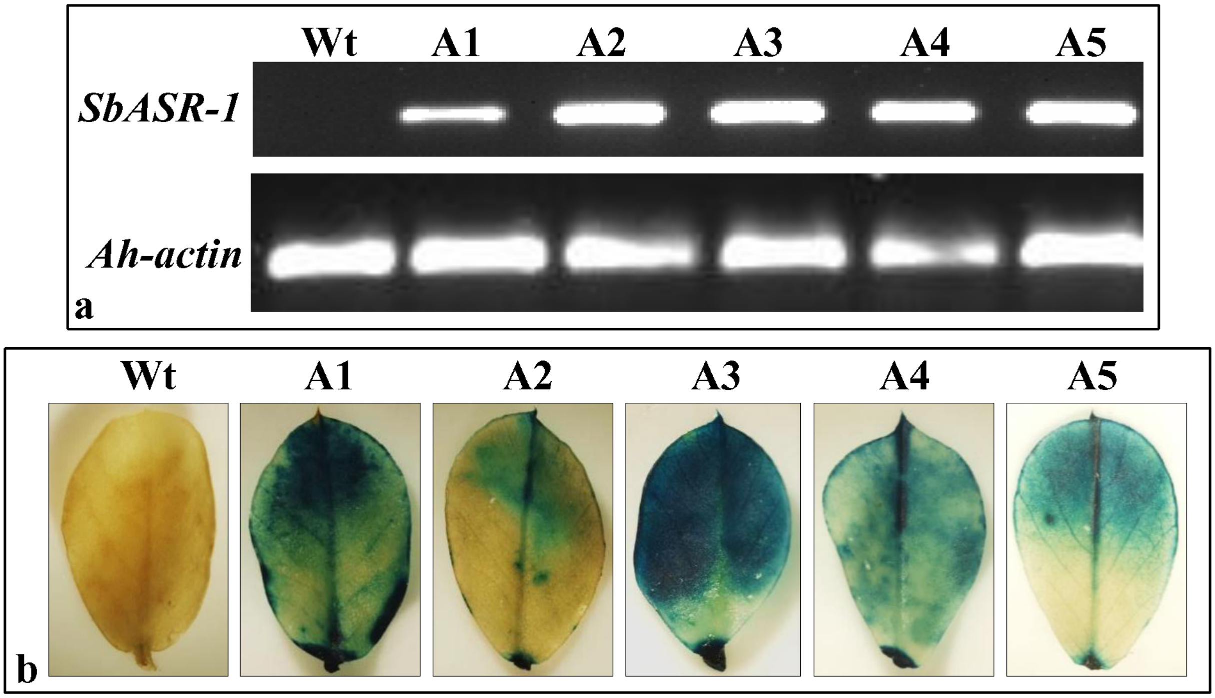

Fig 6. Reverse transcriptase PCR of *SbASR-1* gene in T0 transgenic lines and histochemical GUS assay of leaves from T1 lines. Amplification of 368 bp was observed in all transgenic lines except Wt, whereas *Ah-actin* got amplified (174 bp) in all transgenic lines including Wt (a). Histochemical GUS assay (b) of leaves from Wt plants and T1 transgenic lines.

doi:10.1371/journal.pone.0131567.g006

using the histochemical GUS assay, and three lines (A1, A3, and A4) showing the highest expression (Fig 6B) were selected for further analysis. After 15 days of salinity- and drought-stress treatment, phenotypically better tolerance was observed in the transgenic lines compared to Wt plants (S8 Fig).

Leaf senescence and chlorophyll contents

After 1 week of incubation in NaCl and PEG solution, leaf discs of transgenic lines showed comparatively lower degradation of chloroplasts and stayed greener than Wt plants leaf discs (Fig 7A). There was no significant difference in the Chl a content between Wt plants and transgenic lines under control conditions. The Chl a content in Wt plant discs was reduced to approximately one-third of those under control conditions during NaCl stress and to one-half during osmotic stress, whereas the degree of reduction was lower in transgenic lines (Fig 7B). Transgenic line A3 showed better protection of Chl a from stress conditions than the other two lines. In the case of Chl b, similar results were obtained, showing a two-thirds reduction in Chl b content in Wt plants under both stress treatments, but transgenic lines showed less deterioration (Fig 7C). While estimating the total chlorophyll content, Wt plants showed an approximately 50% reduction under salinity and drought stress compared to the control condition. Transgenic lines A1, A3, and A4 showed only a 20%, 4%, and 40% reduction under salinity (NaCl) stress, respectively, and a 15%, 3%, and 6% reduction under osmotic (PEG) stress, respectively (Fig 7D). Total carotenoid contents also showed the same trend as chlorophyll contents (Fig 7E).

Relative water content, lipid peroxidation, and electrolyte leakage

Water retention ability of the transgenic and Wt plant leaves was compared under control and stress-treatment conditions. Relative water content (RWC) of all transgenic lines was higher under stress conditions, but only A3 and A4 showed significantly higher water content compared to Wt plants leaves during salinity stress. In the case of drought stress, only line A4 showed significantly higher RWC (Fig 8A). Lipid peroxidation of Wt and transgenic lines were compared in leaf tissues by estimating the MDA content—which is produced after lipid peroxidation—that accumulated in leaves. The MDA content was increased abruptly in the Wt plants upon stress treatment relative to the control conditions. It increased by three- and two-fold in salinity and drought stress relative to control conditions, respectively. The MDA content in the transgenic lines also increased slightly, but the increase was not comparable to the Wt plants (Fig 8B). Electrolyte leakage (in %) from leaves of Wt plants and transgenic lines were non-significantly different, and was about 30% in control conditions. Under stress conditions, the leakage in Wt plants increased by 3- and 2.5-fold that of the control in salinity and drought stress conditions, respectively. The electrolyte leakage in transgenic lines was also increased upon stress treatment, but it was significantly lower than that of Wt plants (Fig 8C).

Estimation of proline content

Under control conditions, no significant difference in the proline content was observed between Wt and transgenic lines. Proline content was increased by approximately 17-fold under salinity stress and 7-fold under drought stress conditions in Wt plants, while transgenic lines A1, A3, and A4 showed 12-, 4-, and 2-fold increases in proline content, respectively, under salinity stress. Drought stress-treated transgenic lines showed a slight increase in proline content, in contrast to Wt plants in which it increased by about seven-fold (Fig 8D).

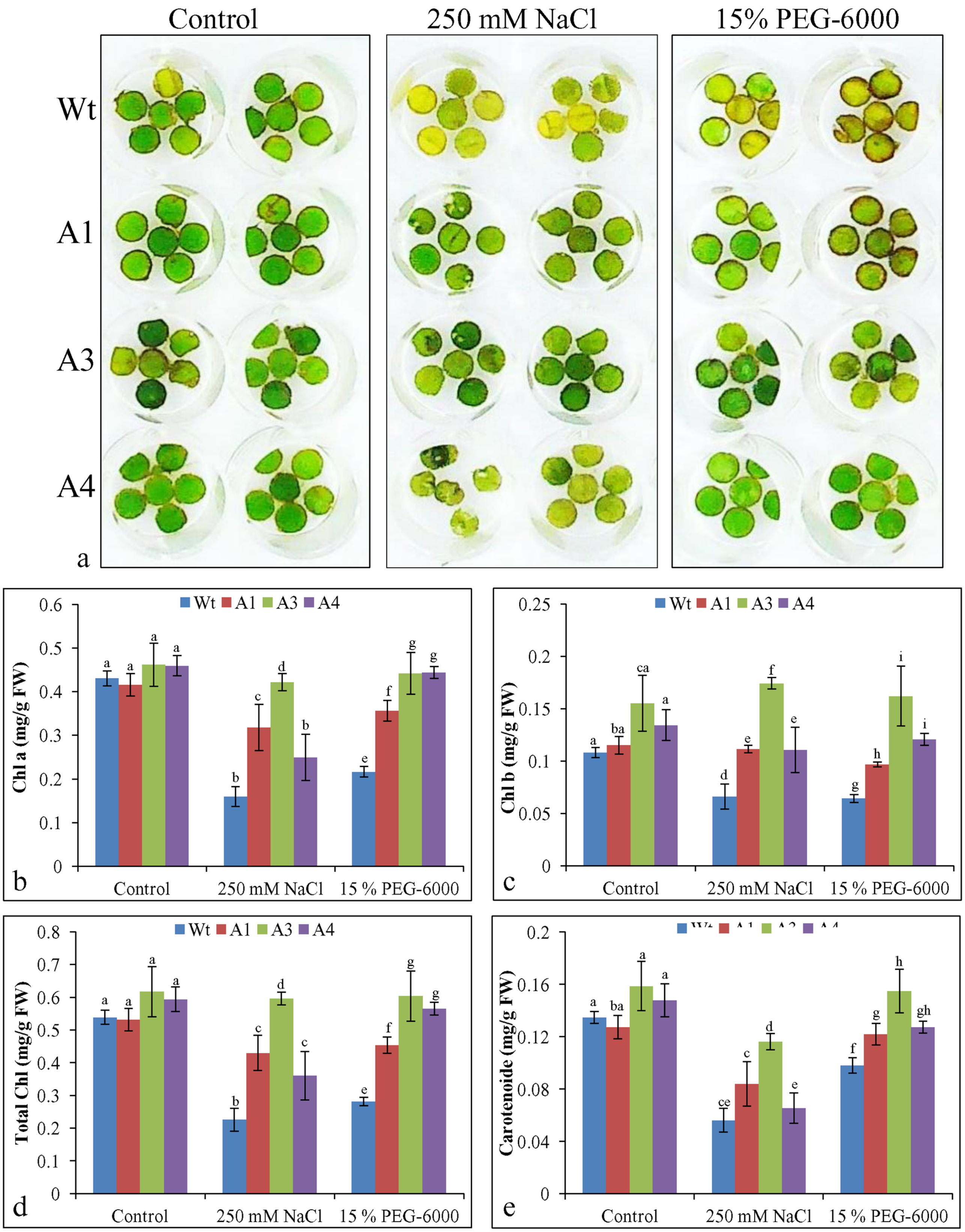

Fig 7. Leaf disc assay and estimation of photosynthetic pigments. Leaf disc assay of transgenic groundnut for salt and drought tolerance (a). Leaf discs of Wt, A1, A3 and A4 transgenic lines respectively were floated in 0 mM, 250 mM NaCl and 15% (w/v) PEG-6000 solution in 0.5x MS-salts for 7-days.

Estimation of (b) chlorophyll a (Chl a), (c) chlorophyll b (Chl b), (d) total chlorophyll and (e) carotenoid content in leaf discs of Wt and transgenic lines. Bars (in b-e) represents means ± SD and values sharing similar letters are non-significant at *P<0.05*.

doi:10.1371/journal.pone.0131567.g007

Total soluble sugar, reducing sugar, and starch content

Total soluble sugars were increased by seven- and three-fold, respectively, under salinity and drought stress conditions in Wt plants as compared to the control condition, but only a slight change was observed in transgenic lines (Fig 9A). The reducing sugar level in Wt plants was increased by 5- and 1.5-fold, respectively, under salinity and drought stress conditions. The transgenic lines also showed an about 1.5-fold increase under stress conditions (Fig 9B). The starch content of the Wt plants increased by three- and two-fold, respectively, under salinity and drought stress conditions compared to control. In contrast, transgenic lines A3 and A4 showed a reduction in starch content under stress. Transgenic line A1 revealed a different result from that of the A3 and A4 lines, and starch content increased slightly under stress (Fig 9C).

In vivo localization of peroxide and superoxide radicals

There were no differences in peroxide localization and free radicals between leaves of Wt plants and transgenic lines under control conditions after staining with DAB and NBT. In contrast,

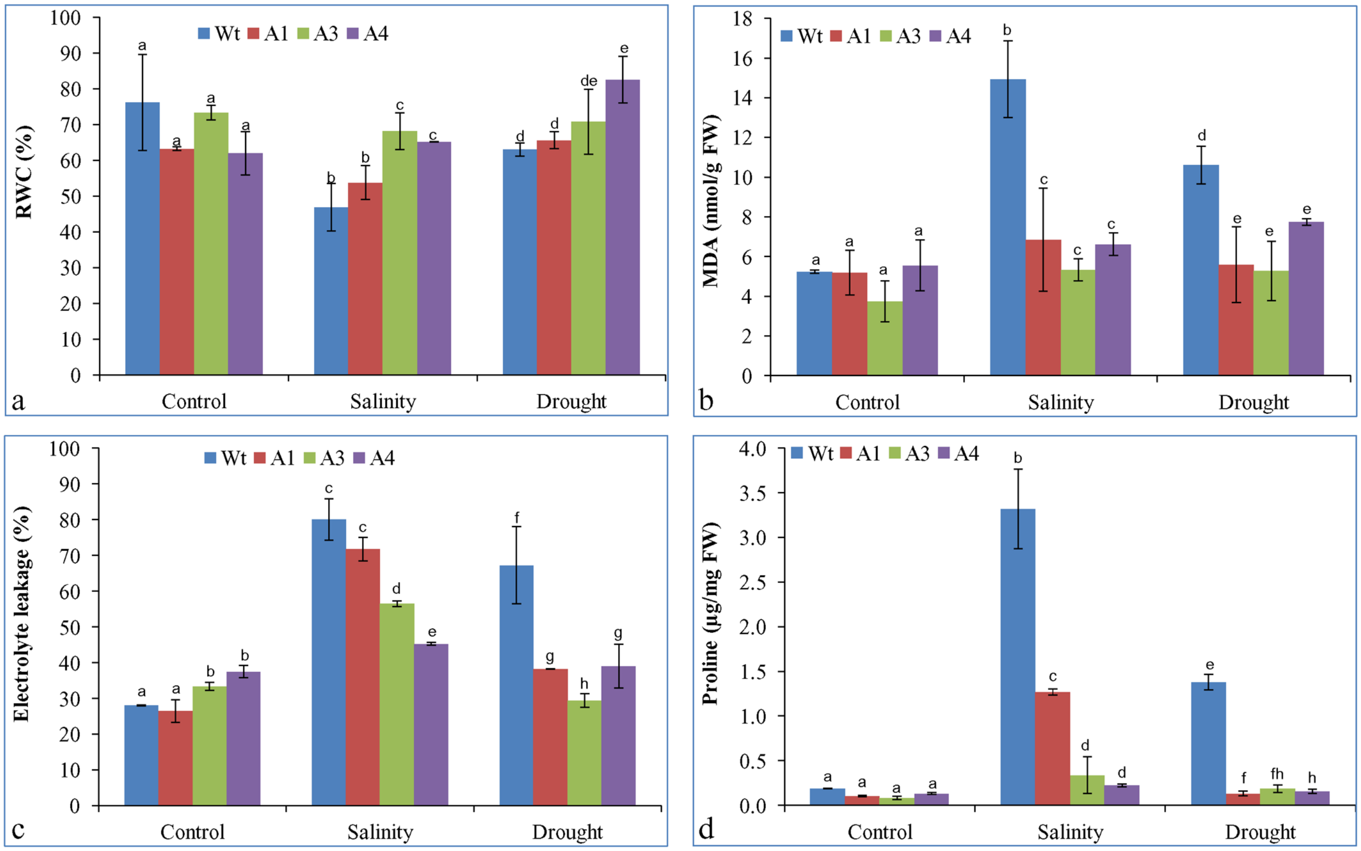

Fig 8. Physiological analysis of transgenic lines. Estimation of (a) RWC, (b) lipid peroxidation, (c) electrolyte leakage and (d) proline content from leaves of Wt plants and transgenic lines under control, salinity (250 mM NaCl) and drought stress conditions. Bars represent means ± SD and values with similar letter are non-significant at *P*<0.05.

doi:10.1371/journal.pone.0131567.g008

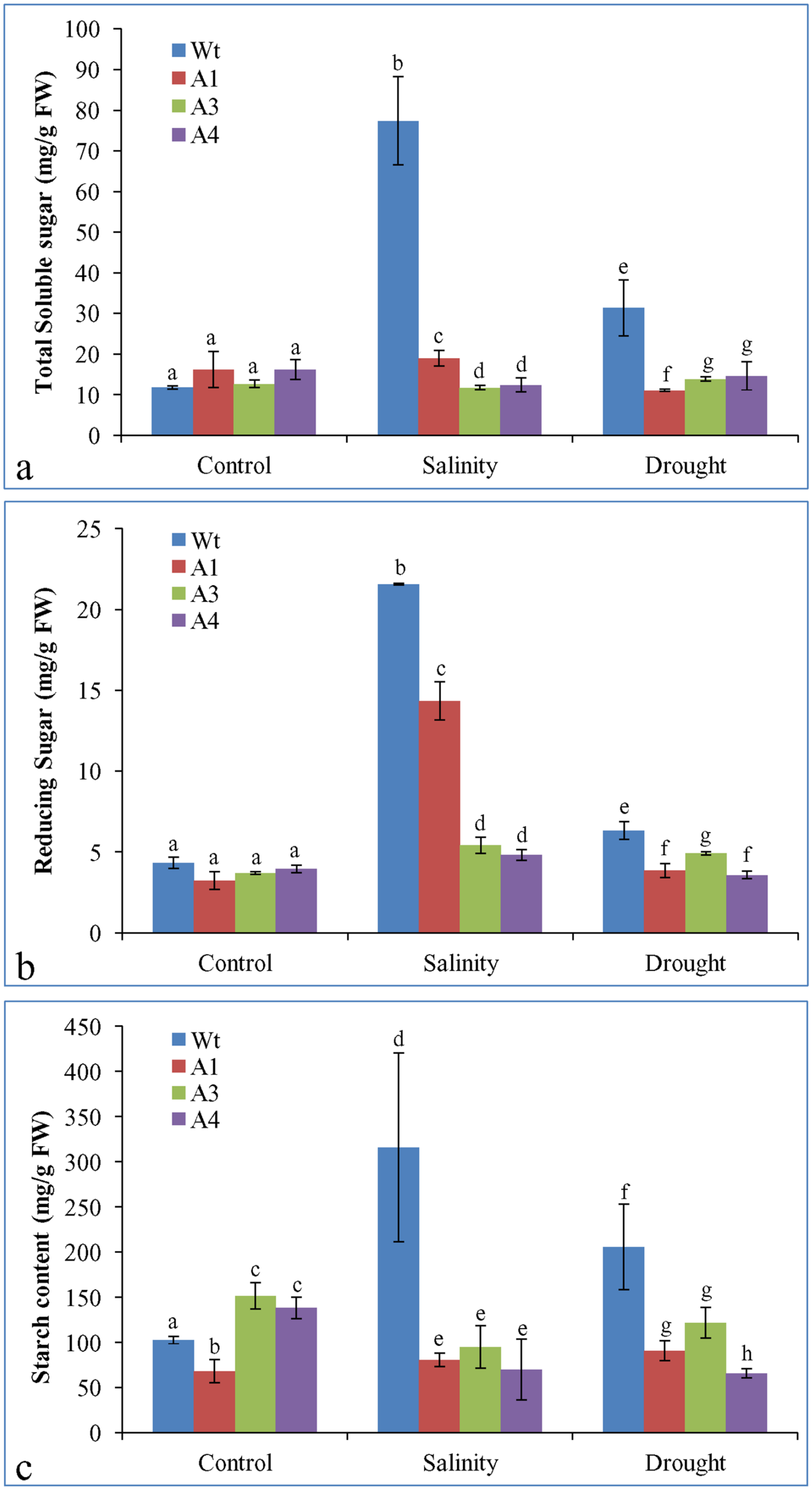

Fig 9. Estimation of Sugar contents. Total soluble sugar (a), reducing sugar (b) and starch content (c) of Wt plants and transgenic lines under control and stress conditions. Bars represent means ± SD and values with similar letter are non-significant at *P<0.05*.

doi:10.1371/journal.pone.0131567.g009

Wt plants leaves exhibited higher levels of brown and blue-colored insoluble precipitate formation than that of transgenic lines under salinity and drought stress (Fig 10). This result demonstrates that Wt plants leaves accumulated more O_2^- and H_2O_2 than that of transgenic lines under stress, confirming that *SbASR-1* helps to minimize stress-induced oxidative stress *in situ*.

Transcript expression analysis of antioxidative enzyme genes

Three genes encoding antioxidative enzymes—ascorbate peroxidase (*APX*), catalase (*CAT*), and superoxide dismutase (*SOD*)—were studied in Wt plants and transgenic line A3 under control and stress conditions (S9 Fig). The expression of *APX* was upregulated by 5.77- and 5.38-fold under salt and drought stress in Wt plants, respectively, while in transgenic line A3, 3.5- and 5.72-fold upregulation was observed under salt and drought stress, respectively. The catalase gene transcript was upregulated by 2.74- and 1.41-fold under salinity and drought stresses, respectively, in Wt plants, but there were inconsistent changes in the expression of *CAT* in the transgenic line. The transcript was downregulated under salinity stress, but upregulated in drought stress conditions. The *SOD* transcript showed slight upregulation under salinity stress, and was downregulated in the drought stress condition in Wt plants. However, there was a uniform trend of upregulation in the transgenic line under both stress conditions.

Discussion

Groundnut (*Arachis hypogaea* L.) is an important oilseed crop that is used for edible oil and as a nutritive supplement. Salinity and drought are the two major abiotic constraints causing significant loss of groundnut productivity worldwide [61]. The traditional breeding program could not be employed efficiently for quality improvement of groundnut due to the tetraploid nature and conserved genome of the cultivated groundnut [34]. Recently, a transgenic approach was used for engineering salt-tolerant groundnut plant by transforming the *SbpAPX* gene, which was cloned from a halophyte [28]. Thus, genetic engineering is one of the comparatively easier approaches to be explored for groundnut quality improvement.

More than 20 years have passed since the discovery of the *ASR-1* gene from tomato and it has been characterized from several glycophytic plants [2], but still the complete functional analysis of any halophytic *ASR-1* has not been reported. Halophytes developed unique ability not only at the morphological, anatomical or physiological level, but they also have different amino acid composition of their protein at molecular level for better adaptation in extreme conditions. They always tend to have high cellular concentration of free amino acids like glutamate, aspartate, proline, glycine etc. which are assisting them in acquiring stress tolerance [18]. The halophytic *SbASR-1* is upregulated in salinity stress conditions, and conferred salt tolerance in transgenic tobacco lines [12]. In the present study, the *SbASR-1* gene was characterized at the genomic and protein function level, and was genetically transformed into a local cultivar of groundnut, GG-20. The salinity and drought stress tolerance of transgenic groundnut was studied in the T1 generation.

The putative promoter region of the *SbASR-1* gene contained important abiotic stress-inducible *cis*-regulatory motifs (Fig 2). The expression of this gene is regulated by an ABA-mediated signaling cascade, which may involve ABRE and MYB binding elements (Table 1). Analysis of *VvMSA* promoter region also showed the presence of several ABA-inducible and DRE motifs [5]. Apart from the stress-inducible motifs, WRKY-binding consensus sequences, pathogen stress-inducible bell-like homeodomain transcription factors, and *cis*-acting motifs involved in sugar repression and phytohormones (such as cytokinin, auxin, GA and SA) regulated pathway were also identified in promoter region of the *SbASR-1* gene (Table 1). The functionality of these motifs was confirmed by previous reports, which demonstrated that the

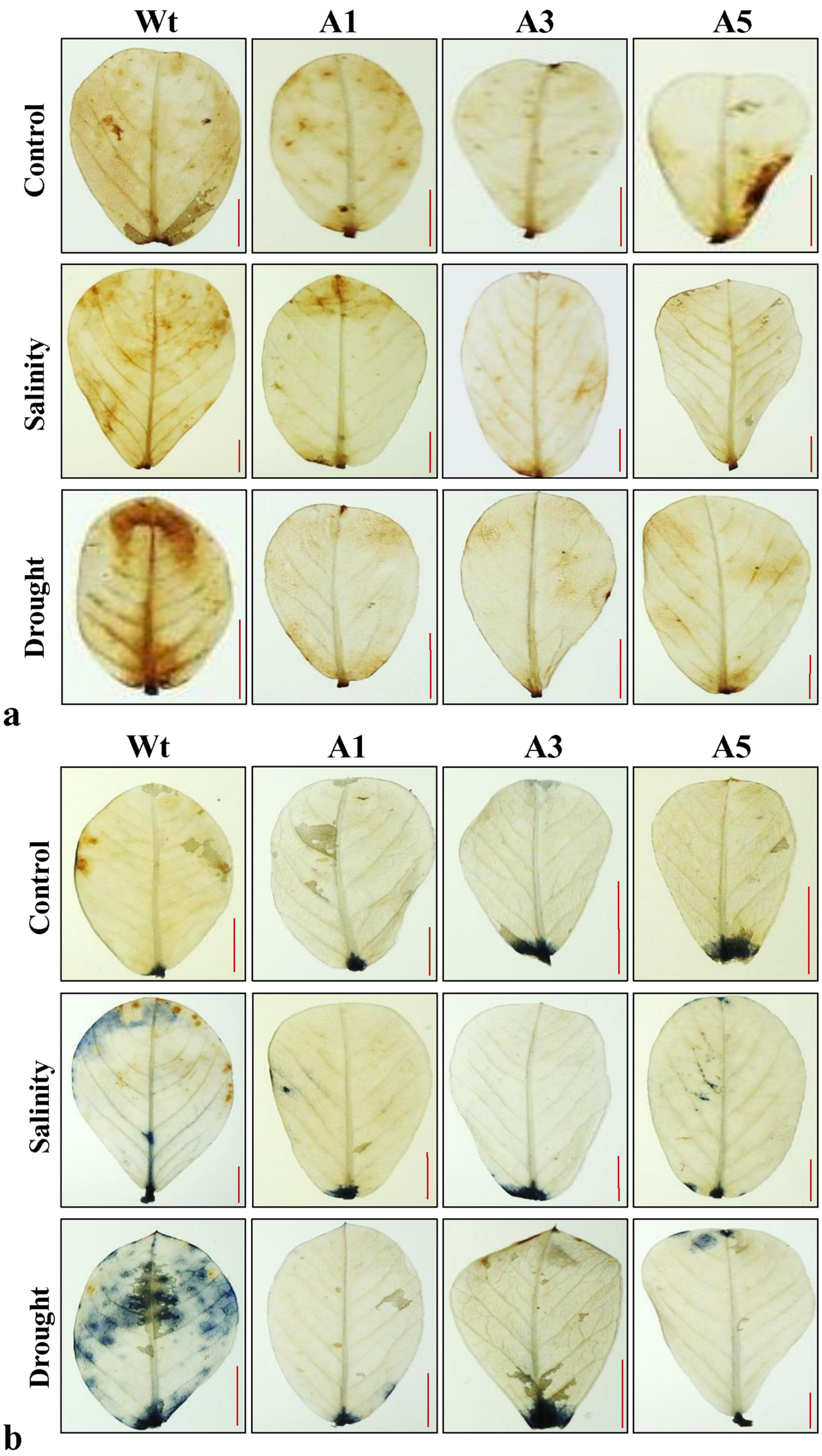

Fig 10. *In-vivo* localisation of peroxide and superoxide free radicals. DAB (a) and NBT (b) staining of Wt plants leaves and transgenic lines leaves. Scale bar represents 5 mm of length.

doi:10.1371/journal.pone.0131567.g010

expression of *ASR* gene transcripts was induced by ABA, salt, drought, PEG-6000, cold, injury, H_2O_2, fruit ripening, Al toxicity, and some biotic factors [2, 4]. ASR-1 also plays a crucial role in regulation of leaf sugar level and mobilization of sugars in other organs and plant growth by regulating GA biosynthesis [4, 8]. This report also supports the presence of A-box and GA-inducible motifs, identified in the *SbASR-1* promoter region.

The genomic organization of the *SbASR-1* gene revealed that only one intron was present in the gene, separating two exons (Fig 2). The intron region is 1611 bp long, which is the largest intron sequence in *ASR* gene family yet reported. The genomic structure of the *ASR* gene family has been reported from the banana, maize, and rice genome. All genes of this family contain either a single intron or are intronless [3, 62–63], which is in agreement with our results. In the case of the *SbASR-1* gene, the intron is several times longer than those in these reports, which might be due to evolutionary adaptation of the gene in *S. brachiata* to cope with harsh environmental conditions, and further study is required to decipher the reason behind it.

At least two paralogs of the *SbASR* gene are present in the *S. brachiata* genome as revealed by Southern hybridization (S2 Fig). The lanes containing DNA digested with *Eco*RI and *Xba*I showed four bands. The *ASR-1* probe sequence shared some part from both the exon (exon I and exon II; Fig 2). It is possible that the presence of a restriction site of these enzymes in the intron region may result in partial hybridization of both the separated exons. The DNA sample digested with *Hind*III showed only two bands, suggesting the presence of two copies of the gene in this family (S2 Fig). The copy number of the *ASR* gene varies in different glycophytic plants. The numbers of paralogs present in the different monocot and dicot plants were reviewed extensively and it was summarized that maize genome contains the highest number of paralogs (nine), banana (four), and tomato (four), whereas the rice genome contains six members [2–3, 62–64]. In contrast, grape *ASR*, also known as *VvMSA*, is present as a single copy in the grape genome [8]. The intrinsically disordered proteins (IDPs), due to presence of high content of disorder promoting amino acid residues ((E, K, R, G, Q, S, P, and A), do not have any fixed conformation. IDPs can undergo extensive post-translational modifications, allowing easier protein–protein interaction for modulation of biological functions because of their structural flexibility [65]. These adaptations of IDPs are for the protection of other cellular proteins under stress conditions and help them in transcriptional regulation of other genes [12]. The *Sb*ASR-1 protein exhibited higher content of disorder promoting amino acid residues (Table 2), confirming that *Sb*ASR-1 is also an IDP. It is supported by the *Sl*ASR-1, which showed a high similarity with *Sb*ASR-1 and protects several proteins from heat induced denaturation and freeze-thaw cycle under *in-vitro* experiments [66]. Dehydration stress responsive dehydrins, ERD10 and ERD14 having high content of these residues, are able to avert the heat-induced aggregation and/or inactivation cellular proteins and enzymes [67]. Glycine residues form zwitterion in solution state, and its abundance in *Sb*ASR-1 protein increased the protein solubility [18] which may assist in better water retention and ROS scavenging. Some of the IDPs of dehydrin family exposed the available side chain H or R of their constituent amino acid residues on the protein surface, which interacts directly with ROS and metal ions to protect cellular proteins from oxidative damage [68]. Role of rice ASR-1 protein as a H_2O_2 scavenger in *in vitro* condition has been experimentally proved [10]. The *Gm*ASR protein was found to chelate the Fe^{3+} ions, and thus may control the hydroxyl radical generation that causes damage to the nucleic acids [11]. Presence of higher glycine content in *Sb*ASR-1 protein may also function in similar way, in which H of the glycine residues interact directly with ROS and metals to prevent cellular damage. Post-translation myristolation of the SOS3 protein is essential for salt stress adaptation through SOS pathway and mutation in the myristoylation sites makes plant hypersensitive to salt stress [69–70]. The highest number of predicted post-translational myristoylation site of *Sb*ASR-1 protein may help in better protein-protein interaction and in

regulating signalling pathways during stress conditions in a better way than that of glycophyte ASR-1.

Heterologous expressed *Sb*ASR-1 protein was found to be approximately 33–35 kDa (S5 Fig), which is higher than the expected theoretical size (21.01 kDa). This abnormal behavior of protein size solely depends on the net charge of protein molecules [7]. To confirm the DNA-binding property of *Sb*ASR-1 protein, which may function as a transcription factor, *in vitro* binding of the protein with DNA probe was tested. The *Sb*ASR-1 protein showed affinity for all the eight DNA probe used in the study and the derived consensus binding sequence is (C/G/A)(G/T)CC(C/G)(C/G/A)(A/T) (Fig 4; S1 and S6 **Figs**). Earlier DNA binding property of tomato ASR-1 protein with recognition sequences C_{2-3}(C/G)A [71] and TCCCCA [50], and rice ASR-1 with (A/T)(A/G)GCCCA consensus-binding site [9] were reported. A similar type of recognition site, GGCCCA (T/A), of the *Os*ASR-5 protein was shown on the promoter region of STAR1 and STAR2 genes, which are involved in aluminum tolerance [72]. The binding affinity of *Sb*ASR-1 with a consensus sequence revealed its ability to bind with all the recognition sites reported earlier for different glycophytic ASR-1. It enables *Sb*ASR-1 to regulate large number of stress responsive genes having these sequences in their regulatory region. The function of *Sb*ASR-1 protein as a transcription factor was further supported by subcellular localization study. Bioinformatics analysis revealed that *Sb*ASR-1 protein sequence contained a nuclear targeting signal similar to *Suaeda liaotungensis* ASR protein [73]. Transient RFP:*Sb*ASR-1 expression analysis clearly support the functionality of the studied NLS sequence and *Sb*ASR-1 was preferentially compartmentalized in the nucleus (Fig 3). Present study confirmed that *Sb*ASR-1 is a nuclear protein which is in line with previous studies on ASR protein of *Suaeda* and wheat [13, 73].

The *SbASR-1* gene was transformed and transgenic groundnut plants were developed (Fig 5). The successful integration of the transgene was confirmed by PCR and Southern hybridization (S7 Fig), and RT-PCR (Fig 6A) showed efficient expression of the transgene. Three transgenic lines (A1, A3, and A4) were selected for further analysis based on higher histochemical GUS expression (Fig 6B). Expression level of transgene in transgenic host plants depends on the site of integration. The Wt plant did not show any expression of the transgene while among transgenic lines, A3 showed higher level of transgene transcript expression than that of others even though similar quantity of cDNA were used in the experiment (Fig 6A).

Chlorophyll content was studied as one of the markers of cellular stress, and it decreases in plants under stress. Previously it was reported that during stress conditions, ROS-scavenging mechanisms failed to cope with the higher rate of ROS generation in the chloroplast, which inhibits the PSII repair system and synthesis of D1 proteins in chloroplasts, and this condition resulted in the loss or degradation of chlorophyll [74]. In the present study, Chl a, Chl b, total chlorophyll, and carotenoid contents were significantly reduced in Wt plants compared to transgenic plants under salinity or drought stress (Fig 7). Similar to this, transgenic tobacco plants overexpressing *TaASR1* and *SbASR-1* exhibited higher chlorophyll content in oxidative stress conditions induced by methyl viologen and salinity stress, respectively [12–13]. Recently, it was observed that rice ASR-1 protein acted as a non-enzymatic antioxidant [10]. Some of the ASR-1 proteins were reported to enhance the expression level of several antioxidative enzymes [11, 13]. Based on these facts, it is presumed that overexpressed *Sb*ASR-1 may have protected the chlorophylls from oxidative damage, and thus transgenic lines maintain higher chlorophyll contents. Carotenoids are reported to be involved in the protection of the photosynthetic machinery by stabilizing thylakoid phospholipids and quenching the excited triplet state of chlorophyll and singlet oxygen under salinity and drought conditions [75]. This may also contribute to the protection of chlorophyll from oxidative damages.

Based on conserved motifs proposed for a special Group 7 of LEA protein family, *Sb*ASR-1 is categorized as a Group 7 LEA protein (S3 Fig). LEA proteins are highly hydrophilic in nature and have strong water-retention ability [6]. The comparative RWC analysis of Wt plants and transgenic groundnut lines exhibited higher relative water content in the transgenic lines than Wt plants under salinity and drought stress (Fig 8A). Similar results were displayed by *SbASR-1*-overexpressing transgenic tobacco lines in salt-treated conditions [12]. Transgenic tobacco plants overexpressing tomato *ASR-1* gene showed a lower rate of water loss than that of Wt plants during NaCl stress [76].

MDA accumulation and electrolyte leakage are common stress markers that measure the degree of injury caused by stress in plants. Stress-induced ROS are responsible for these leakages and MDA formation. MDA is the product of lipid peroxidation caused by ROS [77], whereas electrolyte leakage is efflux of K^+ through ROS-activated cation channels and its counter-ions Cl^-, HPO_4^{2-}, NO_3^-, citrate, and malate [78]. The present study showed higher MDA content and electrolyte leakage in Wt plants than *SbASR-1*-overexpressing transgenic lines under both salinity and drought stress conditions (Fig 8B and 8C). However, under control conditions, transgenic lines and Wt plants exhibited a similar level of MDA and electrolyte leakage. Similar to our findings, transgenic *Arabidopsis* lines overexpressing the banana *ASR-1* gene also showed lower MDA accumulation and electrolyte leakage than Wt plants [79]. The transgenic line A3 showed higher chlorophyll, carotenoid, relative water, MDA content and lower electrolyte leakage, which is directly correlated with the higher level of transgene expression and further conferred better protection of the line than that of other lines under stress conditions.

Proline is a cellular stress marker that accumulates during osmotic stress to maintain the osmotic balance across the membrane. In the present study, the transgenic lines showed lower accumulation of free proline than Wt plants under salinity and drought stress conditions (Fig 8D). Similar results were observed in transgenic tobacco lines overexpressing tomato ASR-1 and *Sb*ASR-1 grown under saline conditions [12, 76]. Lower proline accumulation in leaf tissues of transgenic lines under saline and drought conditions may be due to lower accumulation of Na^+ than that of Wt plants, and potential ROS-scavenging ability of ASR-1 protein, respectively [10, 12, 76].

Total soluble sugar, reducing sugar, and starch content were found to be significantly lower in the transgenic groundnut lines overexpressing *SbASR-1* gene compared to that of Wt plants grown under salinity and drought stress (Fig 9). Silencing of the *ASR-1* gene in tobacco showed higher accumulation of starch, sucrose, and glucose in leaves than Wt tobacco leaves, and revealed the role of ASR-1 in sugar metabolism [4]. Tobacco ASR-1 enhanced the transcript expression of sugar transporters, *i.e.*, hexose transporter, sucrose transporter, and vacuolar glucose transporter proteins [4]. These transporters are involved in the retrieval of glucose and sucrose from leaves to the phloem for mobilization of these nutrients to other organs. Based on our experimental results, we postulate that *Sb*ASR-1 behaves like a transcription factor, may regulates the expression of several sugar transporter proteins, and enhances the tolerance in transgenic plants through better mobilization of nutrients to the root or other non-photosynthetic organs.

In vivo localization of peroxide and superoxide free radicals was carried out, and leaves from Wt and transgenic plants grown under control conditions showed a negligible amount of free radicals (insoluble brown and blue-colored precipitate). In contrast, the leaves from the Wt plants grown under salt- and drought-treated plants showed a higher level of brown and blue-colored precipitate after DAB and NBT staining (Fig 10). This observation indicates the higher level of peroxide and superoxide free radical formation in Wt plants under stress conditions. In agreement with our findings, transgenic tobacco lines overexpressing *TaASR-1* also

showed lower accumulation of H_2O_2 and O_2^- free radicals in seedlings grown under mannitol [13]. Hu *et al*. [13] have shown that *Ta*ASR-1 protein enhanced transcript expression and activity of the antioxidative enzymes, such as catalase and SOD. A Litchi *ASR-1* (*LcASR-1*)-overexpressing transgenic *Arabidopsis* lines also exhibited higher transcript expression of *CAT*, *SOD*, *APX*, and glutathione reductase enzyme genes, and thus participated in better ROS scavenging in transgenic plants [14]. In an *in vitro* experiment, activity of rice ASR-1 protein was found comparable to an antioxidant catalase to scavenge H_2O_2 under stress condition [10]. These reports are consistent with the findings of the present study, and enable an assumption to be made that *Sb*ASR-1 may also be involved in ROS scavenging activity directly as a non-enzymatic antioxidant or indirectly by enhancing the expression of antioxidative enzymes.

To confirm ROS scavenging or transcription factor activity of the *Sb*ASR-1 protein, transcript expression of *APX*, *CAT*, and *SOD* genes were performed in Wt plants and transgenic lines subjected to control or salinity and drought stress (**S9 Fig**). The relative fold-increase of *APX*, *CAT*, and *SOD* transcript expression was higher under salinity stress conditions in Wt plants, whereas it was found to be higher in transgenic lines under drought stress conditions. The major role played by APX and CAT is to detoxify the H_2O_2 into water molecules. Rice ASR-1 could reduce H_2O_2 in water, similar to catalase, in an *in vitro* study [10]. Similar results were found in transgenic tobacco lines overexpressing *TaASR-1* [13]. The ASR-1 protein behaves as a transcription factor, binding the promoter region of several genes directly or by interacting with another transcription factor [8–9, 80]. This function leads to an assumption that *Sb*ASR-1 may act as a transcription factor and regulates the expression of the *SOD* gene to convert superoxide radicals into peroxide, which is further detoxified by *Sb*ASR-1.

Conclusions

The *SbASR-1* gene is 2549 bp in length with a single intron of 1611 bp, which is the largest intron of this family reported so far. There are two copies of this gene in the *S. brachiata* genome. Promoter analyses suggest that the *SbASR-1* gene is stress (abiotic and biotic) and phytohormone inducible. The *Sb*ASR-1 protein has a number of disorder promoting amino acids, glycine residues and N-myristoylation sites, which enable its function more efficiently compared to glycophytic ASR-1 proteins. The *Sb*ASR-1 protein recognizes and binds with the consensus sequence (C/G/A)(G/T)CC(C/G)(C/G/A)(A/T), which are recognized by tomato ASR-1, rice ASR-1 and rice ASR-5 proteins. Biochemical analysis of T1 transgenic groundnut lines showed that overexpressing the *SbASR-1* gene enhances salinity and drought stress tolerance. Increases in transcript expressions of *APX*, *CAT*, and *SOD* were higher in Wt plants under salinity stress, whereas transgenic lines under drought stress showed higher expression of transcripts than Wt plants. It may be concluded that *Sb*ASR-1 enhanced the salinity and drought tolerance of transgenic groundnut significantly by functioning as a transcription factor and LEA protein, and utilizes different pathways of stress tolerance in each type of stress.

Supporting Information

S1 Table. Primers used in the study and PCR conditions.
(DOC)

S2 Table. Blastn analysis of 368 bp *SbASR-1* probe used in southern analysis. It does not show any sequence similarity with the *Arachis hypogaea*.
(XLSX)

S1 Fig. Sequence of DNA probes used for the EMSA experiment. The probe sequences were adopted from the Rom et al. (2006) and their consensus sequence were modified. For probe

ARBS-1 to ARBS-4, consensus sequence were adopted from Rom et al. (2006), while for ARBS-5 to ARBS-8 adopted from Ricardi et al. (2014).
(PPTX)

S2 Fig. Southern hybridization of *S. brachiata* genome to determine copy number of *SbASR-1* gene. Lane PC: Positive control *SbASR-1* cloned vector; Lane E, H and X: Genomic DNA o digested with *Eco*RI, *Hind*III and *Xba*I enzymes, respectively.
(PPTX)

S3 Fig. Amino acid sequence of *Sb*ASR-1 protein denoted in single-letter format. The underlined amino acid sequence shows the conserved domain of ABA/WDS protein super family. Four conserved domains 3, 1, 2 and 5 of LEA Group 7 proteins are displayed in green, red, blue and purple coloured fonts, respectively. The black coloured box in domain 1 represents the DNA binding domain.
(PPTX)

S4 Fig. SDS-PAGE analysis of the 6-Histidine tagged *Sb*ASR-1 recombinant protein expressed in *E.coli* BL21 (DE3) cells. Lane 1, protein standard marker; lane 2–4, BL21 (DE3); lane 5–7, BL21 containing *pET28a*; lane 8–10, BL21 harboring *pET28a*:*SbASR-1* after induction with 1mM IPTG.
(PPTX)

S5 Fig. Purification of recombinant *Sb*ASR-1 protein from *E. coli* BL21 (DE3) cells. Lane 1 showed standard molecular marker of different size. Lane 2–6 have different fractions of elute (E1-E5). A light band of about 70 kDa appears due to dimerization of *Sb*ASR-1 protein.
(PPTX)

S6 Fig. Screening of all eight probes for determining the exact binding site for *Sb*ASR-1 protein. All probes ARSB-1 to ARBS-8 showed clear shifting of band (denoted with blue arrow) which was absent in the lane containing only probe.
(PPTX)

S7 Fig. Molecular confirmation of putative transgenic lines. Amplification of *SbASR-1* gene and Southern hybridization of Wt and different transgenic lines with *SbASR-1* specific probe. Lane M: molecular marker, PC: positive control, Wt: wild type plant (non-transformed) and A1-A5: transgenic lines.
(PPTX)

S8 Fig. Morphological differences in Wt and transgenic lines at control and stress treatment conditions.
(PPTX)

S9 Fig. Transcript expression analysis of three antioxidative enzymes APX, CAT and SOD.
(PPTX)

Acknowledgments

CSIR-CSMCRI Communication No.: PRIS- 127/14. Dr. Santlal Jaiswar is duly acknowledged for providing technical support in the microscopic analysis of localization study.

Author Contributions

Conceived and designed the experiments: AM BJ. Performed the experiments: VT AKC. Analyzed the data: VT AM. Wrote the paper: VT AM.

References

1. Iusem ND, Bartholomew DM, Hitz WD, Scolnik PA (1993) Tomato (*Lycopersicon esculentum*) transcript induced by water deficit and ripening. Plant Physiol 102:1353–1354. PMID: 8278555
2. González RM, Iusem ND (2014) Twenty years of research on *ASR* (ABA-stress-ripening) genes and proteins. Planta 239:941–949. doi: 10.1007/s00425-014-2039-9 PMID: 24531839
3. Virlouvet L, Jacquemot MP, Gerentes D, Corti H, Bouton S, Gilard F, et al. (2011) The *Zm*ASR1 protein influences branched-chain amino acid biosynthesis and maintains kernel yield in maize under water-limited conditions. Plant Physiol 157:917–936. doi: 10.1104/pp.111.176818 PMID: 21852416
4. Dominguez PG, Frankel N, Mazuch J, Balbo I, Iusem N, Fernie AR, et al. (2013) *ASR1* mediates glucose-hormone cross talk by affecting sugar trafficking in tobacco plants. Plant Physiol 161:1486–1500. doi: 10.1104/pp.112.208199 PMID: 23302128
5. Saumonneau A, Laloi M, Lallemand M, Rabot A, Atanassova R (2012) Dissection of the transcriptional regulation of grape ASR and response to glucose and abscisic acid. J Exp Bot 63:1495–1510. doi: 10.1093/jxb/err391 PMID: 22140241
6. Battaglia M, Olvera-Carrillo Y, Garciarrubio A, Campos F, Covarrubias AA (2008) The enigmatic LEA proteins and other hydrophilins. Plant Physiol 148:6–24. doi: 10.1104/pp.108.120725 PMID: 18772351
7. Goldgur Y, Rom S, Ghirlando R, Shkolnik D, Shadrin N, Konrad Z, et al. (2007) Desiccation and zinc binding induce transition of tomato abscisic acid stress ripening 1, a water stress- and salt stress-regulated plant-specific protein, from unfolded to folded state. Plant Physiol 143:617–628. PMID: 17189335
8. Çakir B, Agasse A, Gaillard C, Saumonneau A, Delrot S, Atanassova R (2003) A grape ASR protein involved in sugar and abscisic acid signaling. Plant Cell 15:2165–2180. PMID: 12953118
9. Ricardi MM, González RM, Zhong S, Domínguez PG, Duffy T, Turjanski PG, et al. (2014) Genome-wide data (ChIP-seq) enabled identification of cell wall-related and aquaporin genes as targets of tomato *ASR1*, a drought stress-responsive transcription factor. BMC Plant Biol 14:29. doi: 10.1186/1471-2229-14-29 PMID: 24423251
10. Kim IS, Kim YS, Yoon HS (2012) Rice ASR1 protein with reactive oxygen species scavenging and chaperone-like activities enhances acquired tolerance to abiotic stresses in *Saccharomyces cerevisiae*. Mol Cells 33:285–293. doi: 10.1007/s10059-012-2253-x PMID: 22382682
11. Li RH, Liu GB, Wang H, Zheng YZ (2013) Effects of Fe^{3+} and Zn^{2+} on the structural and thermodynamic properties of a soybean ASR protein. Biosci Biotechnol Biochem 77:475–481. PMID: 23470734
12. Jha B, Lal S, Tiwari V, Yadav S, Agarwal P (2012) The *SbASR*-1 gene cloned from an extreme halophyte *Salicornia brachiata* enhances salt tolerance in transgenic tobacco. Mar Biotechnol 14:782–792. doi: 10.1007/s10126-012-9442-7 PMID: 22639284
13. Hu W, Huang C, Deng X, Zhou S, Chen L, Li Y, et al. (2013) *TaASR1*, a transcription factor gene in wheat, confers drought stress tolerance in transgenic tobacco. Plant Cell Environ 36:1449–1464. doi: 10.1111/pce.12074 PMID: 23356734
14. Liu J, Jia C, Dong F, Wang J, Zhang J, Xu Y, et al. (2013) Isolation of an abscisic acid senescence and ripening inducible gene from litchi and functional characterization under water stress. Planta 237:1025–1036. doi: 10.1007/s00425-012-1820-x PMID: 23223881
15. Ozgur R, Uzilday B, Sekmen AH, Turkan I (2013) Reactive oxygen species regulation and antioxidant defence in halophytes. Funct Plant Biol 40:832–847.
16. Kosová K, Vítámvás P, Urban MO, Prášil IT (2013) Plant proteome responses to salinity stress–comparison of glycophytes and halophytes. Funct. Plant Biol 40:775–786.
17. Flowers TJ, Munns R, Colmer TD (2014) Sodium chloride toxicity and the cellular basis of salt tolerance in halophytes. Ann Bot 115:419–431. doi: 10.1093/aob/mcu217 PMID: 25466549
18. Joseph D, Chakraborty K, Subin CS, Vijayan KK (2013) Halophytes of chenopodiaceae and aizoaceae from South-East coast of India as potential sources of essential nutrients and antioxidants. J Food Nutr Res 1:97–107.
19. Joshi M, Mishra A, Jha B (2012) NaCl plays a key role for in vitro micropropagation of *Salicornia brachiata*, an extreme halophyte. Ind Crops Prod 35:313–316.
20. Mishra A, Joshi M, Jha B (2013) Oligosaccharide mass profiling of nutritionally important *Salicornia brachiata*, an extreme halophyte. Carbohydr Polym 92:1942–1945. doi: 10.1016/j.carbpol.2012.11.055 PMID: 23399241
21. Jha B, Singh NP, Mishra A (2012) Proteome profiling of seed storage proteins reveals the nutritional potential of *Salicornia brachiata* Roxb., an extreme halophyte. J. Agric. Food Chem. 60: 4320–4326. doi: 10.1021/jf203632v PMID: 22494338

22. Mishra A, Patel MK, Jha B (2015) Non-targeted metabolomics and scavenging activity of reactive oxygen species reveal the potential of *Salicornia brachiata* as a functional food. J Funct Foods. 13: 21–31.
23. Jha B, Sharma A, Mishra A (2011) Expression of *SbGSTU* (tau class glutathione S-transferase) gene isolated from *Salicornia brachiata* in tobacco for salt tolerance. Mol Biol Rep 38:4823–4832. doi: 10.1007/s11033-010-0625-x PMID: 21136169
24. Chaturvedi AK, Mishra A, Tiwari V, Jha B (2012) Cloning and transcript analysis of type 2 metallothionein gene (*SbMT-2*) from extreme halophyte *Salicornia brachiata* and its heterologous expression in *E. coli*. Gene 499:280–287 doi: 10.1016/j.gene.2012.03.001 PMID: 22441126
25. Joshi M, Jha A, Mishra A, Jha B (2013) Developing transgenic *Jatropha* using the *SbNHX1* gene from an extreme halophyte for cultivation in saline wasteland. PLoS ONE 8 (8): e71136. doi: 10.1371/journal.pone.0071136 PMID: 23940703
26. Chaturvedi AK, Patel MK, Mishra A, Tiwari V, Jha B (2014) The *SbMT-2* gene from a halophyte confers abiotic stress tolerance and modulates ROS scavenging in transgenic tobacco. PLoS One 9 (10): e111379. doi: 10.1371/journal.pone.0111379 PMID: 25340650
27. Singh N, Mishra A, Jha B (2014) Over-expression of the peroxisomal ascorbate peroxidase (*SbpAPX*) gene cloned from halophyte *Salicornia brachiata* confers salt and drought stress tolerance in transgenic tobacco. Mar Biotechnol 16:321–332. doi: 10.1007/s10126-013-9548-6 PMID: 24197564
28. Singh N, Mishra A, Jha B (2014) Ectopic over-expression of peroxisomal ascorbate peroxidase (*SbpAPX*) gene confers salt stress tolerance in transgenic peanut (*Arachis hypogaea*). Gene 547:119–125. doi: 10.1016/j.gene.2014.06.037 PMID: 24954532
29. Tiwari V, Chaturvedi AK, Mishra A, Jha B (2014) The Transcriptional Regulatory Mechanism of the Peroxisomal Ascorbate Peroxidase (*pAPX*) Gene Cloned from an Extreme Halophyte, *Salicornia brachiata*. Plant Cell Physiol 55:201–217. doi: 10.1093/pcp/pct172 PMID: 24285755
30. Udawat P, Mishra A, Jha B (2013) Heterologous expression of an uncharacterized universal stress protein gene (*SbUSP*) from the extreme halophyte, *Salicornia brachiata*, which confers salt and osmotic tolerance to *E. coli*. Gene 536:163–170. doi: 10.1016/j.gene.2013.11.020 PMID: 24291028
31. Kumari A, Kumar A, Wany A, Prajapati GK, Pandey DM (2012) Identification and annotation of abiotic stress responsive candidate genes in peanut ESTs. Bioinformation 8:1211–1219. doi: 10.6026/97320630081211 PMID: 23275722
32. Dita MA, Rispail N, Prats E, Rubiales D, Singh KB (2006) Biotechnology approaches to overcome biotic and abiotic stress constraints in legumes. Euphytica 147:1–24.
33. Ambede JG, Netondo GW, Mwai GN, Musyimi DM (2012) NaCl salinity affects germination, growth, physiology, and biochemistry of bambara groundnut. Braz J Plant Physiol 24:151–160.
34. Pandey MK, Monyo E, Ozias-Akins P, Liang X, Guimarães P, Nigam SN, et al. (2012) Advances in Arachis genomics for peanut improvement. Biotechnology Advances 30:639–651. doi: 10.1016/j.biotechadv.2011.11.001 PMID: 22094114
35. Sharma KK, Lavanya M (2002) Recent developments in transgenics for abiotic stress in legumes of the semi-arid tropics. JIRCAS Working Report No. 23, pp 61–73 ISSN 1341-710X.
36. Mishra A, Pandey R, Bansal S, Tomar A, Khanna VK, Garg GK (2008) Construction of gene cassette harboring HMW glutenin gene of wheat driven by γ-kafirin promoter of sorghum. Acta Biologica Szegediensis 52:277–282.
37. Mishra A, Tomar A, Bansal S, Khanna VK, Garg GK (2008) Temporal and spatial expression analysis of gamma kafirin promoter from Sorghum (*Sorghum bicolor* L. moench) var. M 35–1. Mol Biol Rep 35:81–88. PMID: 17273894
38. Mishra A, Bansal S, Tomar A, Khanna VK, Garg GK (2009) PCR based isolation and cloning of HMW glutenin gene (s) from wheat (*T. aestivum* var. PBW343) and its fusion with kafirin gene promoter of sorghum. Acta Biologica Szegediensis 53:9–15.
39. Pandey R, Mishra A, Garg GK (2008) Plant promoter driven heterologous expression of HMW glutenin gene (s) subunit in *E. coli*. Mol Biol Rep 35:153–162. PMID: 17380427
40. Bansal S, Mishra A, Tomar A, Sharma S, Khanna VK, Garg GK (2008) Isolation and temporal endospermal expression of γ-kafirin gene of grain sorghum (*Sorghum bicolor* L. moench) var. M 35–1 for introgression analysis of transgene. J Cereal Sci 48:808–815.
41. Singh N, Mishra A, Joshi M, Jha B, (2010) Microprojectile bombardment mediated genetic transformation of embryo axes and plant regeneration in cumin (*Cuminum cyminum* L.). Plant Cell Tiss Org Cult 103:1–6.
42. Joshi M, Mishra A, Jha B (2011) Efficient genetic transformation of *Jatropha curcas* L. by microprojectile bombardment using embryo axes. Ind Crops Prod 33:67–77.

43. Pandey S, Mishra A, Patel MK, Jha B (2013) An efficient method for *Agrobacterium*-mediated genetic transformation and plant regeneration in cumin (*Cuminum cyminum* L.). App Biochem Biotechnol 171:1–9.

44. Banjara M, Zhu L, Shen G, Payton P, Zhang H (2012) Expression of an *Arabidopsis* sodium/proton antiporter gene (*AtNHX1*) in peanut to improve salt tolerance. Plant Biotechnol Rep 6:59–67.

45. Qin H, Gu Q, Kuppu S, Sun L, Zhu X, Mishra N, et al. (2013) Expression of the Arabidopsis vacuolar H^+-pyrophosphatase gene *AVP1* in peanut to improve drought and salt tolerance. Plant Biotechnol Rep 7:345–355.

46. Manjulatha M, Sreevathsa R, Kumar AM, Sudhakar C, Prasad TG, Tuteja N, et al. (2014) Overexpression of a pea DNA helicase (*PDH45*) in peanut (*Arachis hypogaea* L.) confers improvement of cellular level tolerance and productivity under drought stress. Mol Biotechnol 56:111–125. doi: 10.1007/s12033-013-9687-z PMID: 23881361

47. Patil M, Ramu SV, Jathish P, Sreevathsa R, Reddy PC, Prasad TG, et al. (2014) Overexpression of *AtNAC2* (ANAC092) in groundnut (*Arachis hypogaea* L.) improves abiotic stress tolerance. Plant Biotechnol Rep 8:161–169.

48. Qin H, Gu Q, Zhang J, Sun L, Kuppu S, Zhang Y, et al. (2011) Regulated expression of an Isopentenyltransferase gene (*IPT*) in peanut significantly improves drought tolerance and increases yield under field conditions. Plant Cell Physiol 52:1904–1914. doi: 10.1093/pcp/pcr125 PMID: 21920877

49. Higo K, Ugawa Y, Iwamoto M, Korenaga T (1999) Plant *cis*-acting regulatory DNA elements (PLACE) database. Nucleic Acids Res 27:297–300. PMID: 9847208

50. Rom S, Gilad A, Kalifa Y, Konrad Z, Karpasas MM, Goldgur Y, et al. (2006) Mapping the DNA- and zinc-binding domains of ASR1 (abscisic acid stress ripening), an abiotic-stress regulated plant specific protein. Biochimie 88:621–628. PMID: 16387406

51. Tiwari V, Chaturvedi AK, Mishra A, Jha B (2015) An efficient method of *Agrobacterium*-mediated genetic transformation and regeneration in local Indian cultivar of groundnut (*Arachis hypogaea*) using grafting. App Biochem Biotechnol 175: 436–453.

52. Inskeep WP, Bloom PR (1985) Extinction coefficients of chlorophyll a,b in *N*,*N*-dimethylformamide, 80% acetone. Plant Physiol 77:483–485. PMID: 16664080

53. Chamovitz D, Sandmann G, Hirschberg J (1993) Molecular and biochemical characterization of herbicide-resistant mutants of cyanobacteria reveals that phytoene desaturation is a rate limiting step in carotenoid biosynthesis. J Biol Chem 268:17348–17353. PMID: 8349618

54. Barrs HD, Weatherle PE (1962) A Re-Examination of the Relative Turgidity Technique for Estimating Water Deficits in Leaves. Aust J Biol Sci 15:413–428.

55. Hodges DM, DeLong JM, Forney CF, Prange RK (1999) Improving the thiobarbituric acid-reactive-substances assay for estimating lipid peroxidation in plant tissues containing anthocyanin and other interfering compounds. Planta, 207:604–611.

56. Bates LS, Waldern R, Teare ID (1973) Rapid determination of free proline for water stress studies. Plant Soil 39:205–207.

57. Yem EW, Willis AJ (1954) The estimation of carbohydrates in plant extracts by anthrone. Biochem J 57:769–773.

58. Ghose TK (1987) Measurement of cellulase activities. Pure App Chem 59:257–268.

59. Yang S, Wang F, Guo F, Meng J-J, Li X-G, Dong S-T, et al. (2013) Exogenous calcium alleviates photoinhibition of PSII by improving the xanthophyll cycle in peanut (*Arachis Hypogaea*) leaves during heat stress under high irradiance. PLoS ONE 8:e71214. doi: 10.1371/journal.pone.0071214 PMID: 23940721

60. Livak KJ, Schmittgen TD (2001) Analysis of relative gene expression data using realtime quantitative PCR and the $2^{-\Delta\Delta CT}$ method. Methods 25:402–408. PMID: 11846609

61. FAOSTAT (2014) FAO statistics division. http://faostat.fao.org/ (accessed on 20 February 2014).

62. Philippe R, Courtois B, McNally KL, Mournet P, El-Malki R, Le-Paslier MC, et al. (2010) Structure, allelic diversity and selection of *ASR* genes, candidate for drought tolerance, in *Oryza sativa* L. and wild relatives. Theor Appl Genet 121:769–787 doi: 10.1007/s00122-010-1348-z PMID: 20454772

63. Henry IM, Carpentier SC, Pampurova S, Van Hoylandt A, Panis B, Swennen R, et al. (2011) Structure and regulation of the *ASR* gene family in banana. Planta 234:785–798 doi: 10.1007/s00425-011-1421-0 PMID: 21630042

64. Frankel N, Carrari F, Hasson E, Iusem ND (2006) Evolutionary history of the *Asr* gene family. Gene 378:74–83 PMID: 16822623

65. Uversky VN, Dunker AK (2010) Understanding protein non-folding. Biochim Biophys Acta 1804:1231–1264. doi: 10.1016/j.bbapap.2010.01.017 PMID: 20117254

66. Konrad Z, Bar-Zvi D (2008) Synergism between the chaperone-like activity of the stress regulated ASR1 protein and the osmolyte glycine-betaine. Planta 227:1213–1219. doi: 10.1007/s00425-008-0693-5 PMID: 18270732

67. Kovacs D, Kalmar E, Torok Z, Tompa P (2008) Chaperone activity of ERD10 and ERD14, two disordered stress-related plant proteins. Plant Physiol 147:381–390. doi: 10.1104/pp.108.118208 PMID: 18359842

68. Hanin M, Brini F, Ebel C, Toda Y, Takeda S, Masmoudi K (2011) Plant dehydrins and stress tolerance. 6:1503–1509. doi: 10.4161/psb.6.10.17088 PMID: 21897131

69. deJonge HR, Hogema B, Tilly BC (2000) Protein N-myristoylation: critical role in apoptosis and salt tolerance. Sci STKE 2000:pe1–4.

70. Ishitani M, Liu J, Halfter U, Kim C-S, Shi W, Zhu J-K (2000) SOS3 function in plant salt tolerance requires N-myristoylation and calcium binding. Plant Cell 12:1667–1677. PMID: 11006339

71. Kalifa Y, Gilad A, Konrad Z, Zaccai M, Scolnik PA, Bar-Zvi D (2004). The water- and salt-stress-regulated Asr1 (abscisic acid stress ripening) gene encodes a zinc-dependent DNA-binding protein. Biochem. J 381:373–378. PMID: 15101820

72. Arenhart RA, Bai Y, de Oliveira LFV, Neto LB, Schunemann M, dos Santos Maraschin F, et al. (2014) New insights into aluminum tolerance in rice: The ASR5 protein binds the STAR1 promoter and other aluminum-responsive genes. Mol Plant 7:709–721. doi: 10.1093/mp/sst160 PMID: 24253199

73. Hu YX, Yang X, Li XL, Yu XD, Li QL (2014) The *SlASR* gene cloned from the extreme halophyte *Suaeda liaotungensis* K. enhances abiotic stress tolerance in transgenic *Arabidopsis thaliana*. Gene 549:243–251 doi: 10.1016/j.gene.2014.07.071 PMID: 25088570

74. Bartels D, Sunkar R (2005) Drought and Salt Tolerance in Plants. Crit Rev Plant Sci 24:23–58

75. Ramel F, Birtic S, Cuine S, Triantaphylides C, Ravanat JL, Havaux M (2012) Chemical quenching of singlet oxygen by carotenoids in plants. Plant Physiol 158:1267–1278 doi: 10.1104/pp.111.182394 PMID: 22234998

76. Kalifa Y, Perlson E, Gilad A, Konrad Z, Scolnik PA, Bar-Zvi D (2004) Over-expression of the water and salt stress-regulated *ASR1* gene confers an increased salt tolerance. Plant Cell Environ 27:1459–1468

77. Moore K, Roberts LJ (1998) Measurement of Lipid Peroxidation. Free Radical Res 28:659–671

78. Demidchik V, Straltsova D, Medvedev SS, Pozhvanov GA, Sokolik A, Yurin V (2014) Stress-induced electrolyte leakage: the role of K^+-permeable channels and involvement in programmed cell death and metabolic adjustment. J Exp Bot 65:1259–1270 doi: 10.1093/jxb/eru004 PMID: 24520019

79. Dai JR, Liu B, Feng DR, Liu HY, He YM, Qi KB, et al. (2011) *MpASR* encodes an intrinsically unstructured protein and enhances osmotic tolerance in transgenic *Arabidopsis*. Plant Cell Rep 30:1219–1230 doi: 10.1007/s00299-011-1030-1 PMID: 21327389

80. Saumonneau A, Agasse A, Bidoyen M, Lallemand M, Cantereau A, Medici A, et al. (2008) Interaction of grape ASR proteins with a DREB transcription factor in the nucleus. FEBS Lett 582:3281–3287. doi: 10.1016/j.febslet.2008.09.015 PMID: 18804467
